# Development and validation of a measure to assess patient experience of needling of arteriovenous fistulas or grafts for haemodialysis access: the NPREM

**DOI:** 10.1093/ckj/sfaf029

**Published:** 2025-01-28

**Authors:** Currie Moore, Amanda Busby, Rebecca Flanagan, Helen Ellis-Caird, Faizan Awan, Tarsem Paul, Catherine Fielding, Kieran McCafferty, Sabine N van der Veer, Ken Farrington, David Wellsted

**Affiliations:** School of Health and Society, University of Salford; School of Life and Medical Sciences, University of Hertfordshire; School of Life and Medical Sciences, University of Hertfordshire; School of Life and Medical Sciences, University of Hertfordshire; School of Life and Medical Sciences, University of Hertfordshire; Independent; Independent; Research and Innovation and Healthcare of Older People, Nottingham University Hospitals NHS Trust; Renal, Barts Health NHS Trust; Division of Informatics, Imaging and Data Science, School of Health Sciences, Manchester Academic Health Science Centre, University of Manchester, Manchester, UK; Renal, East North Hertfordshire NHS Trust; School of Life and Medical Sciences, University of Hertfordshire

**Keywords:** cannulation, haemodialysis, needling, patient reported experience measure, vascular access

## Abstract

**Background:**

Needling is a key step in haemodialysis. Research suggests that needling experience is sub-optimal; however, no validated measure exists to inform improvements. We addressed this by developing the Needling Patient Reported Experience Measure (NPREM).

**Methods:**

We used mixed methods and co-production. All participants were adults with working fistulas/grafts from eight UK kidney centres. Phase 1 involved developing concepts and items: in interviews (*n* = 41), we explored patients’ needling experience and identified key aspects of needling using thematic analysis. This informed the 98-item NPREM(v0.1). Phase 2 was piloting the measure: cognitive interviews (*n* = 16) assessed face validity. Items were amended or removed, yielding a 48-item NPREM(v0.2). A pilot survey (*n* = 183) examined initial psychometric properties. NPREM(v0.2) showed good internal consistency (Cronbach's alpha = 0.95). Review of analyses resulted in a 35-item NPREM(v0.3). Phase 3 involved evaluating the measure's dimensionality, validity and reliability: patients (*n* = 468) completed the NPREM(v0.3), Vascular Access Quality of Life (VASQoL), EuroQol 5-Dimension-5-Level (EQ-5D-5L) and Patient Activation Measure (PAM), with a sub-set completing a follow-up NPREM (*n* = 99). Items were evaluated with 28 items retained in the NPREM(v1.0). Confirmatory factor analysis confirmed a unidimensional model fit (comparative fit index = 0.899). Validity of the NPREM(v1.0) was good [convergent: VASQoL (*r *= 0.60) and overall experience (*r *= 0.79); divergent: EQ-5D (*r *= –0.31), EQ-5D visul analogue scale (*r *= 0.24) and PAM (*r *= 0.17)]. Test–retest scores were strongly correlated (*r *= 0.88), demonstrating high reliability. Known-groups validity was demonstrated between centre scores [range 5.21 (standard deviation 1.20) to 5.94 (0.75)].

**Conclusion:**

The NPREM measures patient experience of needling for haemodialysis. It offers kidney services a means of assessing needling experience, informing patient-focused clinical and service improvements.

## Graphical Abstract

**Graphical Abstract:**
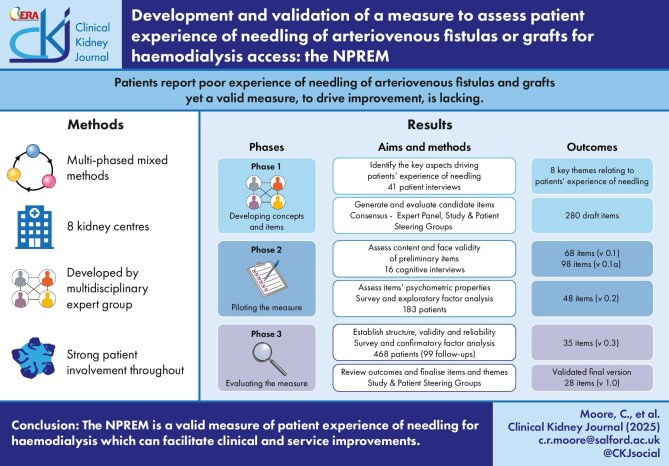

KEY LEARNING POINTS **What was known:**

- Reliable access to the vascular system is vital for patients receiving haemodialysis, and arteriovenous fistulas or grafts provide the safest and most effective route.
- Patients consistently rate their experience of needling of their fistulas or grafts as poorer than their experiences of most other areas of kidney care.
- There is currently no validated measure to assess patient experience of needling in sufficient detail. Availability of such a measure will facilitate clinical and system improvements in this area.

**This study adds:**

- The Needling Patient Reported Experience Measure (NPREM) is the first validated measure which assesses patients’ experience of needling of arteriovenous fistulas or grafts for haemodialysis. It is a patient-centred measure, robustly and rigorously developed with patients for patients.
- The NPREM is a publicly available, 28-item measure that provides a comprehensive view of patients’ experience of needling. It covers five themes of care (Communicating with the Team, My Fistula/Graft and Needling, Steps in Needling, Working Together, My Personal Experience) and Overall Needling Experience.
- The main aspect of kidney care related to variation in overall NPREM scores was the kidney centre providing care.

**Potential impact:**

- The NPREM can be used as part of routine clinical practice to improve care of individual patients or to audit patient care at service-level as part of local quality improvement initiatives; it may also be used as an outcome measure in vascular access or needling research.
- The NPREM may also provide the basis of national dataset benchmarking of patient experience of needling; this would allow a better understanding of what drives differences in patients’ experience of needling between centres.
- Used in these ways, the NPREM will increase focus on this important topic and facilitate joined up care and communication between patients and kidney teams, leading to better needling practices; further work is required to implement the measure.

## INTRODUCTION

Arteriovenous fistulas and grafts, considered the most cost-effective forms of access and associated with the lowest complication rates and mortality [1, 2], must have needles inserted each dialysis session, commonly referred to as ‘needling’ by patients [3].

Patients consistently report lower scores for their experience of needling than other aspects of their care [4]. In qualitative research, needling is associated with pain and anxiety [5–8]. Furthermore, patients who experience poor needling may avoid fistulas and grafts and rely on central lines [6, 9–11].

Using validated measures to routinely monitor patients’ experience of care is evident across all levels of healthcare and informs quality improvements [12–15]. The routine collection of the UK Kidney Patient Reported Experience Measure (PREM) led to patient-centred initiatives, delivered locally and nationally, to improve kidney care [4].

Although the UK Kidney PREM includes an item on needling, it is limited in scope (e.g. only applicable to people on incentre and satellite haemodialysis, focussed on pain). The 9-item Dialysis Fear of Injection Questionnaire identifies patients with a fear of needling [16] and captures personal reactions to needling (e.g. restlessness) but not broader aspects of needling. In the field of vascular access, measures exist that assess patient satisfaction or the impact of access on their lives [17–20]; however, they do not focus on needling. Measures that reflect outcomes important to patients, such as needling problems and impact on wellbeing, are required to progress clinical trials and research [21]. Despite evidence that needling is sub-optimal, valid ways of measuring patients’ overall experience of needling are lacking.

This study aimed to develop and validate a Needling Patient Reported Experience Measure (NPREM) to be used to inform service improvements and as an outcome in research. Co-produced with people with lived experience of needling [22], this programme of research sought to:

(i) better understand adult patients’ experiences of needling
(ii) examine how needling experience could be reliably and validly assessed

## MATERIALS AND METHODS

### Approach

**Figure 1:**
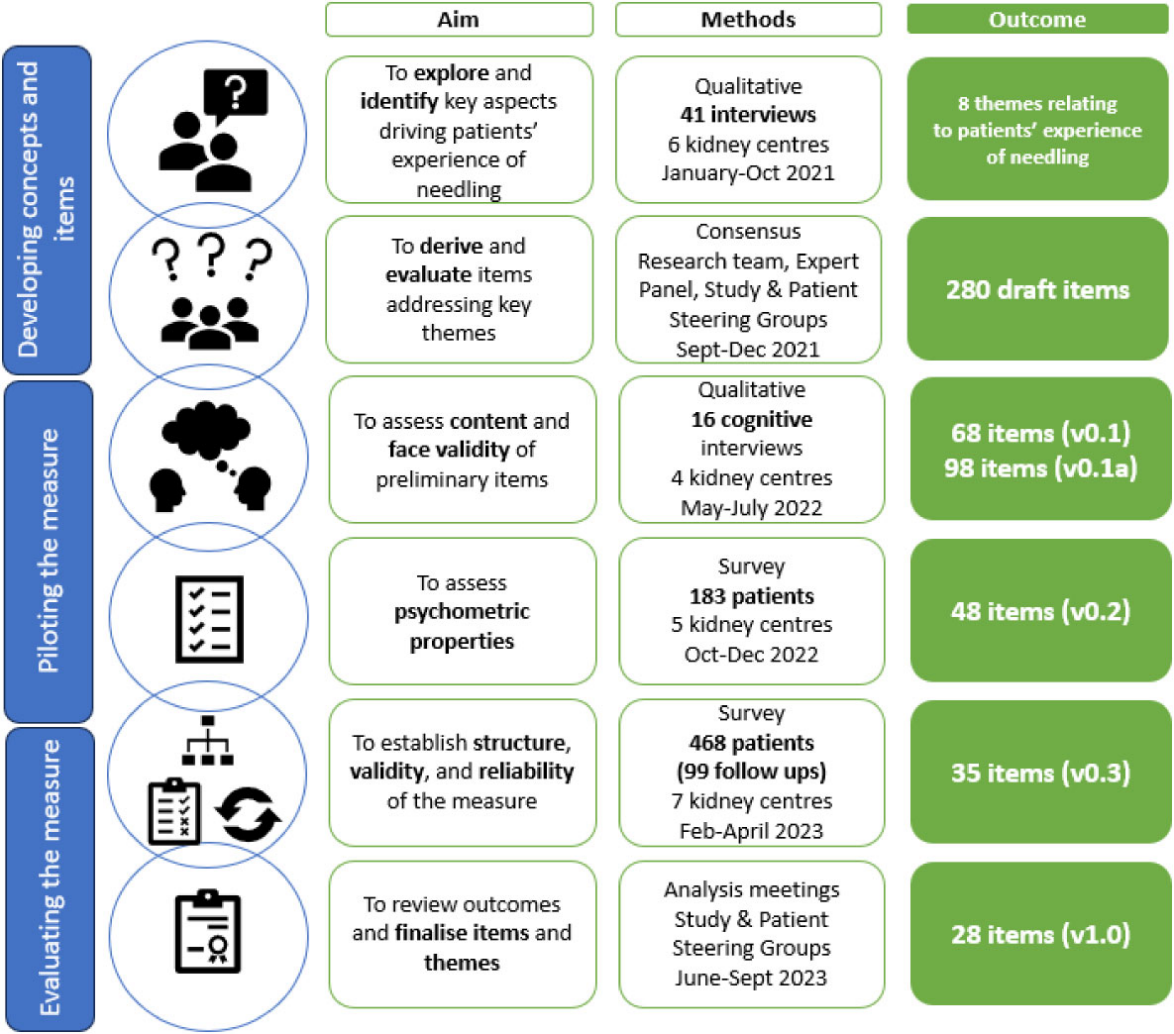
Phases of development and evaluation. Adapted from Boateng *et al*. (2018) ‘Best practices for developing and validating scales for health, social, and behavioral research: a primer’ (p. 2) [23]*.*

We followed established recommendations for scale development (Fig. 1) [23]. Supporting documents providing additional details are available [Supplementary data, Supporting Material 1 (SM1)]. Study- and Patient-Steering-Groups, including healthcare professionals, researchers and people with lived experience of needling (Supplementary data, SM2), were integral throughout [24].

### Ethical approval

This study received favourable ethical opinion from the UK Health Research Authority and National Health Service (NHS) Research Ethics Committees (Cornwall-Plymouth, Ref. No. 17/NW/0501).

### Setting and participants

In all phases participant eligibility criteria were: >18 years, receiving haemodialysis, working fistula/graft, and >3 months since starting dialysis. Eight NHS kidney centres in England participated in the study across phases (Supplementary data, SM3). In qualitative phases, written consent was obtained. In quantitative phases, return of questionnaires implied consent.

### Phase 1: developing concepts and items

We aimed to identify pertinent aspects to needling, rooted in the patients’ experience, and to generate potential items. A full account of Phase 1 is reported in a corresponding publication [25].

#### Data collection

Qualitative methods, utilizing both unstructured and semi-structured interviews, provided authentic descriptions of patients’ experience of needling [26]. We used purposive sampling to ensure inclusion of a range of needling experiences. Interviews were conducted in English, Gujarati or Urdu via telephone or online, lasting on average 61 (range 12–115) min. All were audio-recorded and transcribed verbatim.

#### Analysis

Thematic analysis, employing both inductive and deductive coding, enabled an in-depth examination of patients’ needling experiences, revealing common themes [26, 27]. Inductive codes captured personal nuances, while deductive codes highlighted shared experiences. We managed the data with QDA Miner (v5). After unstructured interviews, preliminary themes were identified. Themes were refined through semi-structured interviews, including cultural relevance checks, until consensus was reached on the overall findings. Developing codes and concepts were assessed in research team meetings and with the Patient-Steering-Group following an iterative process.

#### Item generation

We identified putative items addressing the concepts developed from interview analysis, the literature and other relevant measures, aiming for 50–70 preliminary items. An expert panel (*n* = 10), consisting of patients, clinicians, methodologists and researchers, met online and then assessed item relevance via an online survey (Qualtrics, 1 = not relevant, 4 = highly relevant). Each item's content validity index (i-CVI) and modified Kappa was calculated [28]. The Patient- and Study-Steering-Groups approved preliminary items.

### Phase 2: piloting the measure

We conducted initial item assessment in the target population by establishing face and content validity and evaluating items’ psychometric properties.

#### Phase 2a: testing items’ face and content validity

##### Data collection

Using cognitive interviewing, we examined how patients interpreted the items and formulated responses. ‘Think aloud’ and ‘verbal probes’ [29] were used during the interviews, conducted via telephone or video-call. Interviews lasted on average 51 (range 30–75) min and were audio-recorded.

##### Analysis

A coding framework [30, 31] facilitated identification of issues. Each item was reviewed by the team and Patient-Steering-Group with those considered suitable selected by consensus for inclusion.

#### Phase 2b: pilot survey

##### Data collection

Five centres recruited patients by issuing paper NPREM(v0.2) packs over 7 weeks, each targeting 30–50 participants (*N* = 150–250). Survey data enabled the evaluation of scale characteristics, provided data on item reliability, and supported identification of poorly performing items. A 7-point Likert scale captured responses with ‘Not Applicable’ and ‘Don't Know’ options.

##### Analysis

Assuming a limited number of factors (maximum 3) and moderate fit (7%–10% change in R^2^), 150 participants were required to provide study power greater than 1 – β = 0.80 for α = 0.05. Analysis included psychometric evaluation of each item across key variables using descriptive statistics and by analysing item response distributions and response option usage. Cronbach's alpha assessed internal reliability, α > 0.90 considered sufficient but α > 0.95 desirable [32]. Exploratory factor analysis with varimax rotation examined the preliminary factor structure. Results were considered alongside inter-item correlations, aiming to reduce item number [33]. The number of underlying dimensions was assessed by examining eigenvalues >1 and inspecting scree plots to determine the last substantial decline in magnitude of eigenvalues [34]. To investigate potential item order effects, three versions of the NPREM (A, B, C) were distributed randomly to test response variation on two items: painfulness of needling [Test 1 (T1)] and overall experience (T2).

### Phase 3: evaluating the measure's dimensionality, validity and reliability

This phase followed the same overall procedures as the pilot with a larger patient population to assess the NPREM's dimensionality, validity and reliability.

#### Data collection

Seven kidney centres distributed NPREM packs to potential participants over a 10-week period. Surveys were also available for online completion (Qualtrics). A subset of participants completed follow-up NPREM and Change of Circumstances form 2–4 weeks later (surveys linked by unique codes). The NPREM pack included 20 sociodemographic and clinical questions, 6 general questions and 3 additional questionnaires to enable NPREM construct validity assessment: Vascular Access Specific Quality of Life (VASQoL) [19], EuroQol 5-Dimension-5-level (EQ-5D-5L including overall health item) [35] and Patient Activation Measure (PAM) [36].

#### Analysis

Sample size was selected pragmatically; assuming up to three factors with 18 degrees of freedom, α = 0.05 and 1 – β = 0.80 with sensitivity to evaluate a 3% change in R^2^, 473 participants were required. Allowing for attrition, seven centres aimed to recruit approximately 60–70 patients each (*N* = 420–490), with 10–15 completing follow-ups (*N* = 70–105). Data processing followed the same approach as the pilot. Exploratory factor analysis evaluated the factor structure [33]. Findings were reviewed by item and theme groupings, and headings finalized. Confirmatory factor analysis confirmed the robustness and internal consistency of item selection. Missing data was handled using the maximum likelihood with missing values approach [37]. Model fit statistics included the comparative fit index (CFI < 0.9 acceptable) [38], χ^2^ (lower values relative to degrees of freedom indicating better model fit) [39], and root mean square error of approximation (RMSEA; <0.05 considered good, 0.05–0.08 acceptable, 0.08–0.1 marginal, >0.1 poor) [40, 41]. Sensitivity analyses were undertaken excluding items not applying to all participants (e.g. buttonholing, pain relief usage) to ensure group selection did not influence internal consistency.

##### Convergent and divergent validity

Correlations (Pearson's) between the NPREM scale score and other scales were used to assess construct validity. Convergent validity was evaluated (cut off *r *> 0.50) [42] for the VASQoL and Overall Experience item (Q30). Divergent validity was assessed (cut off *r *< 0.40) [42] for EQ-5D-5L, EQ-5D overall health item and PAM.

##### Test–retest reliability

Assuming α = 0.05 and correlation coefficient *r *= 0.7 [43], a sample size *n* = 101 for test–retest provided a precision of 0.2 standard deviations (SD) for *r*. Two-way mixed-effect analysis of variance model (estimating random effects for participants and fixed effects for time) intra-class coefficient correlations (ICCs) for absolute agreement [44] and Pearson correlation coefficients were used to evaluate test–retest reliability, separately calculated for those reporting changes and those reporting no changes in circumstances between completion of the two surveys.

##### Known-groups validity

Variables where differences in needling experience were anticipated were grouped and scale scores compared as following: gender, age (<65 years/65+ years), needler group (nurse-led/self), needling activeness (active/not active), haemodialysis location (unit/home, centre/satellite), access technique (rope ladder/buttonhole), access type (fistula/graft), first access (yes/no), pain relief used (none/yes) and by centre. T-tests and regression models were used to compare groups, with *P < *.05 considered statistically significant. Differences >0.7 (10% of the scale) were deemed meaningful between groups. Quantitative data analyses were performed using Stata (v18).

## RESULTS

Table 1 reports participant characteristics across all phases.

**Table 1:** Participant characteristics across study phases.

		Phase 1 *n* = 41	Phase 2a *n* = 16	Phase 2b *n* = 183	Phase 3 *n* = 468	Test–retest *n* = 99
Gender [*n* (%)]	Male	25 (60.9)	10 (62.5)	115 (63.2)	308 (67.1)	64 (64.6)
Age [years, mean (SD)]		60 (16.7)	48 (14.4)	64.39 (13.9)	65.85 (13.8)	64.24 (14.4)
Ethnicity [*n* (%)]	Asian	7 (17.1)	2 (12.5)	12 (6.9)	46 (10.0)	6 (6.1)
	Black	6 (14.6)	4 (25.0)	30 (17.2)	50 (10.9)	7 (7.1)
	White	25 (60.9)	10 (62.5)	129 (74.1)	348 (76.0)	81 (82.7)
	Mixed/other	3 (7.3)	0 (0)	3 (1.7)	14 (3.0)	2 (2.0)
Access type [*n* (%)]	Fistula (vs graft)	33 (80.4)	13 (81.3)	169 (93.9)	422 (95.3)	88 (92.6)
First access [*n* (%)]	Yes		13 (81.3)	141 (80.1)	341 (76.1)	69 (72.6)
Technique [*n* (%)]	Buttonhole	10 (24.3)	8 (50.0)	36 (20.1)	80 (18.1)	26 (26.5)
	Rope ladder			61 (34.1)	146 (33.1)	33 (33.7)
	Area puncture			57 (31.8)	141 (32.0)	26 (26.5)
	Not sure (RL/AP)			25 (14.0)	74 (16.8)	13 (13.3)
HD location [*n* (%)]	Main renal unit	20 (49)	9 (56.3)	91 (50.6)	202 (44.9)	47 (48.5)
	Satellite unit	18 (43.8)	2 (12.5)	82 (45.5)	206 (45.8)	36 (37.1)
	Home	3 (7.3)	5 (31.2)	2 (1.1)	30 (6.7)	12 (12.4)
	Missing			5 (2.8)	12 (2.7)	2 (2.1)
HD routine [*n* (%)]	≤3 times per week	37 (90.4)	11 (68.8)	178 (99.0)	416 (95.8)	89 (90.8)
	>3 times per week	4 (9.6)	5 (31.2)	2 (1.0)	18 (4.2)	9 (9.2)
Time on dialysis [months, median (IQR)]		37 (19, 72)	60 (24, 120)	36 (18, 60)	36 (18, 65)	40 (21, 83)
Access location [*n* (%)]	Dominant arm			58 (31.9)	129 (29.3)	29 (30.5)
	Non-dominant arm			123 (67.6)	309 (70.1)	64 (67.4)
	Leg			1 (0.5)	3 (0.7)	2 (2.1)
Age of access [months, median (IQR)]		29 (20, 60)	54 (18, 70)	30 (14, 60)	27 (14, 60)	30 (15, 72)
Needler [*n* (%)]	Healthcare staff (always)	35 (85.4)	8 (50.0)	168 (94.9)	425 (90.8)	85 (85.9)
	Self (at least sometimes)	6 (14.6)	7 (43.7)	9 (5.1)	43 (9.2)	14 (14.1)
	Other	0 (0)	1 (6.3)	0 (0.0)	0 (0.0)	0 (0.0)
Pain relief used [*n* (%)]	None	32 (78.0)	13 (81.2)	136 (76.0)	318 (72.4)	62 (64.6)
	Numbing cream	8 (19.5)	2 (12.5)	34 (19.0)	100 (22.8)	27 (28.1)
	Lignocaine spray	0 (0)	0 (0)	1 (0.6)	11 (2.5)	2 (2.1)
	Lignocaine injection	1 (2.4)	1 (6.3)	9 (5.0)	16 (3.6)	5 (5.2)
Pain relief provider [*n* (%)]	Unit			21 (47.7)	71 (49.7)	20 (54.1)
	GP			20 (45.5)	60 (42.0)	13 (35.1)
	Self-bought			3 (6.8)	12 (8.4)	4 (10.8)

Personal, sociodemographic, and clinical characteristics varied across study phases, with Phases 1 and 2a informing data to be collected in Phase 2b and 3. In Phase 1 and 2a only ‘Needling Technique—Buttonhole’ was reported as it was distinguishable from rope ladder and area puncture.

AP, area puncture; GP, general practice; HD, haemodialysis; IQR, interquartile range; RL, rope ladder.

### Phase 1: developing concepts and items

In total, 41 patients participated in this phase. After the first 24 interviews, we identified 11 key aspects of needling [25]. In the subsequent 17 interviews, we checked these themes and assessed cultural relevance with four non-English speakers. Themes were refined and agreed between the research team, Patient- and Study-Steering-Groups resulting in eight initial themes (Supplementary data, SM4).

#### Item generation

The research team and Patient-Steering-Group generated approximately 280 initial items addressing key themes identified in the interviews and literature, which were sequentially amended and reduced. Of these, 52 received consensus for inclusion in the cognitive interviews with seven items not reaching consensus. The expert panel assessed these seven items’ relevance (Supplementary data, SM5) and reviewed the remaining 52 items [24]. Their assessment suggested two items retained, one revised and four excluded. Considering the expert panel's feedback, the research team and Patient-Steering-Group re-examined and edited items (e.g. changing all items to present tense, clarifying concepts), resulting in the 68-item preliminary NPREM(v0.1) (Fig. 1).

### Phase 2: piloting the measure

#### Phase 2a: testing items’ face and content validity

In cognitive interviews, 16 patients, purposively selected from four centres, assessed preliminary NPREM items. Following the first set of cognitive interviews, NPREM(v0.1) items were amended or added (Supplementary data, SM6), resulting in a 98-item NPREM(v0.1a) (Fig. 1). Using the coding framework, we identified issues and amended them accordingly (Supplementary data, SM7). The research team and Patient-Steering-Group refined the items and converted suitable items to statements, resulting in a 48-item NPREM(v0.2) (Fig. 1).

#### Phase 2b: pilot survey

The NPREM(v0.2) (Supplementary data, SM8) was given to 244 patients, of which 183 viable responses were included in analysis [63% male, mean age 64.4 years (SD 13.9), 74% White, access via fistula 93.9%].

##### Item response profile

Item means ranged from 2.65 to 6.76 (scale 1–7), with *n* = 45 (94%) item means above 5.0 (Supplementary data, SM9). For two items, ‘Don't Know’ was selected by >10% of respondents, with five items rated ‘Not Applicable’ by >10%. As commonly observed for PREMs, participants tended to use the high end of the scale, with a ceiling effect shown in eight items; scale point 7 selected by >80% of participants, 1–3 responses totalling <10% in *n* = 33 (68.8%) items.

##### Overall scale analysis

Exploratory factor analysis indicated that needling experience was a unidimensional construct, with good internal consistency (Cronbach's α = 0.95) and moderate to strong inter-item correlations (Supplementary data, SM9). Although three additional factors had eigenvalues >1 (Supplementary data, SM10), these were >10 points smaller than the primary factor, suggesting a single factor was most appropriate, as also indicated in the scree plot (Supplementary data, SM11). Multifactor models were examined, with no improvement in model fit or retained items. Sensitivity analyses showed no differences in factor structure when imputing missing values.

##### Order effect

An order effect was seen in relation to the overall experience question (T2) (Supplementary data, SM12), with mean responses significantly lower when placed at the beginning rather than the end of the measure. This indicated that the full experience of needing is not considered when at beginning; therefore, T2 was placed at the end of the measure. No statistically significant order effects were observed with the pain item (T1).

##### Changes to NPREM(v0.2) informed by pilot analysis

Of the 48 items tested, 14 displayed significant psychometric issues and were excluded. Five items, with poor psychometric properties yet clinically important, were retained separately as ‘service’ items (Supplementary data, SM5). After further review, one item was added, three rephrased, and themes re-examined with items re-allocated to two identified themes, communication and involvement, resulting in a 35-item NPREM(v0.3) (Fig. 1).

### Phase 3: evaluating the measure's dimensionality, validity and reliability

The NPREM(v0.3) was circulated to 711 patients, of which 468 viable responses were included [67% male, mean age 66 years (SD 14), 76% White, 95% access using fistula]. To assess reliability follow-up surveys were sent to 206 patients, with 99 viable responses included.

#### Item response profile

Most items had means >5.0, reflecting high endorsement; however, all SDs were >1.0 (range 1.11–2.31) reflecting response variation (Table 2). ‘Don't Know’ was selected by >10% of respondents in three items and ‘Not Applicable’ was selected by >10% for four items. No items had scale point 7 selected by >80% of participants, and 19 items scale points 1–3 were selected by <10%, a reduction in ceiling effect from the pilot survey.

**Table 2:** Phase 3: item response profile and internal consistency of the NPREM(v0.3).

				1		2		3		4		5		6		7									
NPREM (v0.3)	*N*	Mean (SD)	Median	*N*	%	*N*	%	*N*	%	*N*	%	*N*	%	*N*	%	*N*	%	Don't know	Not applicable	Missing	Correlations >0.60	Item-test	Item-rest	Inter-item	Cronbach's alpha
Q1	454	4.65 (1.62)	5	18	4.0	37	8.1	56	12.3	82	18.1	96	21.1	113	24.9	52	11.5	1	3	10		0.441	0.393	0.352	0.947
Q2	453	5.19 (1.62)	6	14	3.1	26	5.7	33	7.3	60	13.2	76	16.8	143	31.6	101	22.3	0	6	9		0.521	0.477	0.349	0.947
Q3	446	6.20 (1.40)	7	12	2.7	7	1.6	9	2.0	19	4.3	36	8.1	83	18.6	280	62.8	2	10	10	Q5	0.590	0.549	0.346	0.946
Q4	444	5.69 (1.61)	6	16	3.6	17	3.8	17	3.8	32	7.2	59	13.3	118	26.6	185	41.7	9	6	9		0.606	0.568	0.345	0.946
Q5	351	5.90 (1.60)	7	13	3.7	12	3.4	10	2.8	18	5.1	37	10.5	80	22.8	181	51.6	89	14	14	Q3, Q15, Q20, Q23	0.676	0.641	0.343	0.945
Q6	454	4.53 (2.17)	5	66	14.5	34	7.5	55	12.1	60	13.2	50	11.0	54	11.9	135	29.7	5	2	7		0.456	0.410	0.352	0.947
Q7	451	6.10 (1.44)	7	9	2.0	8	1.8	18	4.0	28	6.2	41	9.1	73	16.2	274	60.8	3	5	9		0.681	0.649	0.342	0.945
Q8	419	6.08 (1.41)	7	7	1.7	12	2.9	12	2.9	21	5.0	42	10.0	90	21.5	235	56.1	8	19	22		0.647	0.614	0.343	0.945
Q9	436	5.89 (1.65)	7	18	4.1	15	3.4	12	2.8	28	6.4	42	9.6	87	20.0	234	53.7	5	8	19	Q14, Q15	0.623	0.585	0.344	0.945
Q10	432	5.30 (1.94)	6	31	7.2	22	5.1	35	8.1	43	10.0	42	9.7	86	19.9	173	40.0	14	4	18		0.486	0.438	0.350	0.947
Q11	444	6.25 (1.24)	7	7	1.6	3	0.7	7	1.6	31	7.0	29	6.5	97	21.8	270	60.8	2	4	18	Q12, Q14, Q20, Q23	0.716	0.683	0.341	0.945
Q12	436	6.31 (1.11)	7	3	0.7	1	0.2	12	2.8	19	4.4	38	8.7	97	22.2	266	61.0	3	12	17	Q11, Q14, Q15, Q20	0.751	0.720	0.340	0.944
Q13	401	5.69 (1.78)	6	18	4.5	21	5.2	16	4.0	34	8.5	36	9.0	76	19.0	200	49.9	38	11	18		0.703	0.674	0.341	0.945
Q14	422	6.18 (1.29)	7	4	0.9	8	1.9	10	2.4	30	7.1	31	7.3	92	21.8	247	58.5	11	17	18	Q9, Q11, Q12, Q15, Q18, Q20, Q23	0.740	0.709	0.339	0.944
Q15	318	5.86 (1.60)	7	13	4.1	8	2.5	9	2.8	19	6.0	44	13.8	65	20.4	160	50.3	48	69	32	Q5, Q9, Q12, Q14, Q18, Q20, Q23	0.717	0.690	0.341	0.945
Q16	421	5.11 (2.17)	6	53	12.6	22	5.2	33	7.8	28	6.7	47	11.2	58	13.8	180	42.8	2	18	27	Q29	0.672	0.640	0.342	0.945
Q17	437	5.60 (1.54)	6	9	2.1	13	3.0	25	5.7	54	12.4	63	14.4	104	23.8	169	38.7	2	3	26		0.702	0.672	0.341	0.945
Q18	413	6.32 (1.39)	7	14	3.4	5	1.2	7	1.7	9	2.2	24	5.8	69	16.7	285	69.0	19	6	30	Q14, Q15, Q20, Q23	0.679	0.647	0.342	0.945
Q19	437	5.17 (2.14)	6	56	12.8	19	4.3	22	5.0	41	9.4	47	10.8	62	14.2	190	43.5	0	3	28		0.588	0.549	0.345	0.946
Q20	403	6.08 (1.47)	7	8	2.0	13	3.2	10	2.5	22	5.5	46	11.4	60	14.9	244	60.5	9	27	29	Q5, Q11, Q12, Q14, Q15, Q18, Q23, Q30	0.745	0.718	0.340	0.944
Q21	420	5.99 (1.64)	7	17	4.0	12	2.9	11	2.6	30	7.1	34	8.1	59	14.0	257	61.2	19	11	18		0.607	0.570	0.345	0.946
Q22	434	6.26 (1.42)	7	13	3.0	3	0.7	17	3.9	16	3.7	22	5.1	68	15.7	295	68.0	2	18	14		0.651	0.616	0.344	0.945
Q23	436	6.31 (1.31)	7	9	2.1	7	1.6	7	1.6	20	4.6	21	4.8	80	18.3	292	67.0	1	15	16	Q5, Q11, Q14, Q15, Q18, Q20, Q30	0.707	0.677	0.341	0.945
Q24	414	5.34 (1.99)	6	34	8.2	22	5.3	27	6.5	40	9.7	36	8.7	75	18.1	180	43.5	21	13	20		0.553	0.513	0.347	0.946
Q25	448	5.73 (1.59)	6	12	2.7	14	3.1	25	5.6	40	8.9	50	11.2	109	24.3	198	44.2	0	6	14		0.449	0.403	0.352	0.947
Q26	442	6.48 (1.26)	7	11	2.5	5	1.1	7	1.6	8	1.8	16	3.6	55	12.4	340	76.9	2	7	16		0.494	0.448	0.350	0.947
Q27	442	5.18 (1.92)	6	36	8.1	24	5.4	31	7.0	38	8.6	59	13.3	112	25.3	142	32.1	5	5	16		0.651	0.617	0.343	0.945
Q28	444	5.76 (1.59)	6	11	2.5	15	3.4	25	5.6	40	9.0	42	9.5	107	24.1	204	45.9	1	3	20		0.574	0.534	0.346	0.946
Q29	425	5.25 (1.99)	6	38	8.9	22	5.2	31	7.3	34	8.0	43	10.1	93	21.9	164	38.6	4	21	18	Q16 ,Q30	0.717	0.688	0.340	0.945
Q30	444	5.97 (1.26)	6	3	0.7	7	1.6	11	2.5	39	8.8	62	14.0	121	27.3	201	45.3	4	1	19	Q20, Q23, Q29	^a^	^a^	^a^	^a^
S1	432	4.73 (2.23)	5	63	14.6	36	8.3	39	9.0	34	7.9	49	11.3	65	15.0	146	33.8	7	12	17		0.477	0.431	0.350	0.947
S2	337	5.42 (2.31)	7	54	16.0	14	4.2	7	2.1	13	3.9	17	5.0	37	11.0	195	57.9	53	53	25		0.445	0.399	0.350	0.947
S3	217	5.47 (1.91)	6	18	8.3	8	3.7	7	3.2	23	10.6	23	10.6	42	19.4	96	44.2	39	189	23		0.514	0.472	0.347	0.946
S4	376	5.66 (1.36)	6	5	1.3	14	3.7	12	3.2	30	8.0	54	14.4	158	42.0	103	27.4	44	14	34		0.343	0.293	0.355	0.948
S5	180	5.41 (2.04)	6	20	11.1	3	1.7	12	6.7	12	6.7	20	11.1	27	15.0	86	47.8	44	206	36		0.517	0.478	0.347	0.946

Responses captured using a 1 to 7 Likert scale with labels at endpoints with ‘Don't Know’ and ‘Not Applicable’ options. Higher scores reflect positive needling experience.

a Overall item (Q30) excluded from NPREM scale score.

#### Exploratory factor analysis

##### Dimensionality

**Figure 2:**
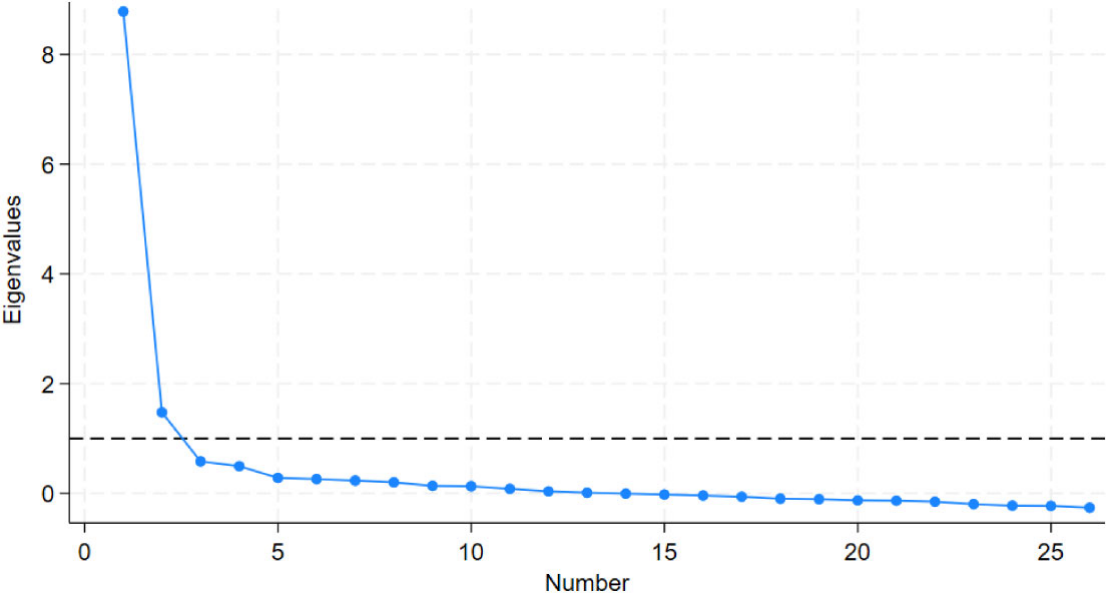
Phase 3: scree plot of eigenvalues of NPREM(v0.3, exploratory factor analysis). Includes data from 447 participants with ‘Don't know’ and ‘Not applicable’ or missing responses mean imputed.

Overall, the NPREM showed good internal consistency (Cronbach's α = 0.94) and moderate to strong inter-item correlations (Table 2). Exploratory factor analyses (*n* = 447) indicated that needling experience remained a unidimensional construct, with one dominant factor (Table 3, Fig. 2). Sensitivity analyses demonstrated that missing data had no effect on the overall scale structure.

**Table 3:** Phase 3: eigenvalues of the first six factors for the NPREM(v0.3), exploratory factor analysis.

Factor	Eigenvalue	Proportion of variance	Cumulative variance
Factor 1^a^	8.942	0.797	0.797
Factor 2	1.490	0.133	0.930
Factor 3	0.609	0.054	0.984
Factor 4	0.487	0.043	1.027
Factor 5	0.274	0.024	1.052
Factor 6	0.262	0.023	1.075

a The high eigenvalue for factor 1 suggests a single factor model consisting of all questions.

##### Changes to NPREM survey

On review, seven items were excluded (three to be collected alongside sociodemographic information), eight minor changes, and two changed theme (Supplementary data, SM8). Theme groupings were reframed, reducing the number from eight to five (Communicating with the Team, Working Together, My Fistula/Graft and Needling, Steps in Needling, My Personal Experience). Following revisions, the final NPREM(v1.0) consisted of 27 items, plus one overall needling experience item (Fig. 1, Table 4).

**Table 4:** NPREM(v1.0) and response labels by theme.

Items by theme		Response labels
Communicating with the team		
Q8	I am involved as much as I want to be in decisions about my needling	Not at all—Completely
Q11	Problems during needling are managed well	Never—Always
Q13	My opinions about needling are taken seriously by the dialysis team	Strongly disagree—Strongly agree
Q18	I have problems communicating with the dialysis team about my needling	Always—Never
Q21	I feel able to tell the dialysis team if something doesn't feel right	Strongly disagree—Strongly agree
My fistula/graft and needling		
Q5	I worry about how long my fistula/graft will keep working	All the time—Not at all
Q12	I have concerns that current needling practices are harmful to my fistula/graft	Strongly agree—Strongly disagree
Q20	There are things about my fistula/graft that make it difficult to needle	Strongly agree—Strongly disagree
Steps in needling		
Q2	I experience problems when the needles are inserted	Always—Never
Q15	I experience problems due to the positioning of the needles once they are inserted	Always—Never
Q17	My fistula/graft is assessed before the needles are placed	Never—Always
Q23	I experience problems when the needles are removed	Always—Never
Q25	My buttonhole scabs are removed with as little pain as possible	Never—Always
Q26	I get the support I need when new buttonhole sites are formed	Strongly disagree—Strongly agree
Q27	The pain relief that I use works well	Strongly disagree—Strongly agree
Working together		
Q3	I trust the dialysis team when it comes to my needling	Not at all—Completely
Q6	My needling is rushed	Always—Never
Q7	I feel that the dialysis team needling me show empathy	Never—Always
Q10	My needling is done in a way that makes me feel safe	Never—Always
Q14	I worry about who will be available to needle me	Always—Never
Q19	The dialysis team put me at ease during needling	Never—Always
My personal experience		
Q1^a^	Overall, how painful is needling?	Not at all painful– Extremely painful
Q4	My needling experience has improved over time	Strongly disagree—Strongly agree
Q9	My frame of mind affects my needling experience	Always—Never
Q16	I am nervous before needling	Always—Never
Q22	My needling experience varies greatly from session to session	Strongly agree—Strongly disagree
Q24	Previous bad experiences of needling still affect how I feel about my needling	Strongly agree—Strongly disagree
Overall needling experience		
Q28	How would you rate your overall needling experience?	Worst it can be—Best it can be

Items are numbered by their recommended order, however items Q2–27 may be presented in any order. Responses use a 1 to 7 Likert scale with labels at endpoints and ‘Don't Know’ and ‘Not Applicable’ also options. Higher scores indicate positive needling experience.

a Q1 reversed scored.

#### Confirmatory factor analysis

##### Dimensionality

A total of 447 cases had sufficient data for inclusion in confirmatory factor analyses with one factor. Inclusion of all items provided a moderate model fit (CFI = 0.823, Table 5). Allowing item covariance within themes improved model fit (CFI = 0.899). Sensitivity analyses confirmed that model fit was unaffected by the removal of items not applying to all patient groups (e.g. buttonhole access, pain relief), with CFIs between 0.898 and 0.905 if items were allowed to covary.

**Table 5:** Phase 3: results from confirmatory factor analysis and sensitivity analyses of the NPREM(v1.0).

	*N*	CFI	χ^2^	df	RMSEA	RMSEA 95% CI
Confirmatory factor analysis						
All items included						
Uncorrelated	447	0.823	1173	324	0.077	0.072–0.081
>MI 40 correlated	447	0.864	972	321	0.067	0.063–0.072
Items in the same theme correlated	447	0.899	745	260	0.065	0.059–0.070
Sensitivity analyses						
Buttonhole item (S5) removed						
Uncorrelated	447	0.822	1148	299	0.080	0.075–0.085
>MI 40 correlated	447	0.863	946	296	0.070	0.065–0.075
Items in the same theme correlated	447	0.898	725	241	0.067	0.062–0.073
Buttonhole (S5) and new site (Q15) items removed						
Uncorrelated	447	0.831	1027	275	0.078	0.073–0.083
>MI 40 correlated	447	0.861	889	273	0.071	0.066–0.076
Items in the same theme correlated	447	0.905	645	222	0.065	0.060–0.071
Pain relief (S3) item removed						
Uncorrelated	447	0.822	1143	299	0.080	0.075–0.085
>MI 40 correlated	447	0.864	942	296	0.070	0.065–0.075
Items in the same theme correlated	447	0.899	721	241	0.067	0.061–0.072
Buttonhole (S5), new site (Q15) and pain relief (S3) items removed						
Uncorrelated	447	0.829	1002	252	0.082	0.076–0.087
>MI 40 correlated	447	0.860	864	250	0.074	0.069–0.080
Items in the same theme correlated	447	0.905	619	203	0.068	0.062–0.074

MI, modification indices; df, degrees of freedom; 95% CI. 95% confidence interval.

##### Convergent and divergent validity

The NPREM(v1.0) scale correlated strongly with the VASQoL (*r *= 0.60, *P *< .0001) and with the Overall Experience item (*r *= 0.79, *P *< .0001), providing evidence of convergent validity. There were weak correlations with the EQ-5D-5L (*r *=* *–0.31, *P *< .0001), the EQ-5D overall health (*r *= 0.24, *P *< .0001) and PAM (*r *= 0.17, *P *= .0003), confirming divergent validity.

##### Test–retest reliability

Retest surveys estimated for 87 respondents, of which 45 indicated no change in circumstances, 25 indicated one change and 17 indicated more than one change (Table 6). NPREM and Overall Experience scores for those not experiencing changes were strongly correlated (NPREM: ICC = 0.87, *r *= 0.89; Q30: ICC = 0.76, *r *= 0.76) with marginally weaker correlations in those experiencing changes (NPREM: ICC = 0.87, *r *= 0.86; Q30: ICC = 0.72, *r *= 0.73), indicating stable scale scores over time irrespective of changes in circumstance.

**Table 6:** Phase 3: test–retest reliability of the NPREM(v1.0).

	Test		Re-test			
	*N*	Mean (SD)	*N*	Mean (SD)	r	Intra-class correlation, ICC (95% CI)
NPREM scale score						
All participants	87	5.53 (1.16)	87	5.56 (1.14)	0.88	0.88 (0.83 to 0.92)
Change or event between test and retest						
No	45	5.87 (0.97)	45	5.97 (0.80)	0.89	0.87 (0.78 to 0.93)
Yes^a^	42	5.17 (1.24)	42	5.12 (1.28)	0.86	0.87 (0.76 to 0.93)
Q30 (Overall item)						
All participants	86	5.88 (1.33)	86	5.76 (1.48)	0.76	0.76 (0.65 to 0.83)
Change or event between test and retest						
No	45	6.27 (1.12)	45	6.16 (1.11)	0.76	0.76 (0.60 to 0.86)
Yes^a^	41	5.46 (1.43)	41	5.32 (1.71)	0.73	0.72 (0.54 to 0.84)

a Changes to treatment *n* = 8, hospital stay *n* = 6, health deteriorated *n* = 7, major life event *n* = 14, specific negative staff interaction *n* = 6, fistuloplasty *n* = 5, hospitalisation due to fistula/graft *n* = 4, stent *n* = 2, surgical procedure *n* = 6, any other factor affecting needling *n* = 2, longer wait than usual between arriving and dialysing *n* = 15, anything else *n* = 5.

95% CI, 95% confidence interval.

##### Known-group validity

Small variations in NPREM(v1.0) scores were apparent by age group (<65 years, mean 5.55, SD 1.03; 65+ years, mean 5.79, SD 1.00; *P *= .016; Table 7). However, scores varied significantly between centres with means ranging from 5.21 (SD 1.20) to 5.94 (SD 0.75), a range of 0.73 (10.4% of scale range). This provides evidence that the NPREM is sensitive to group differences, demonstrating its known-groups validity.

**Table 7:** Phase 3: differences in needling experience by groups.

	*N*	Mean	SD	*P*-value
Gender				
Male	280	5.77	1.02	.070
Female	139	5.57	1.00	
Age				
<65 years	179	5.55	1.03	.016
65+ years	248	5.79	1.00	
Needling				
Nurse	388	5.70	1.03	.752
Self	39	5.64	0.85	
Active in needling				
Active	356	5.68	1.03	.498
Not active	71	5.77	0.95	
Home vs centre HD				
ICHD	383	5.70	1.03	.998
HHD	28	5.70	0.80	
Centre vs satellite HD				
Main Unit	188	5.76	0.88	.265
Satellite	192	5.65	1.14	
Access technique				
Rope ladder	330	5.65	1.06	.116
Buttonhole	76	5.85	0.75	
Access type				
Fistula	387	5.72	1.02	.021
Graft	19	5.17	0.93	
First access				
Yes	313	5.71	1.01	.536
No	96	5.64	1.05	
Pain relief				
None	288	5.73	1.03	.192
Yes	121	5.58	1.01	
Centre				
A	55	5.38	0.95	[ref]
B	84	5.83	1.02	.010
C	66	5.94	0.75	<.001
D	71	5.88	0.87	.005
E	43	5.59	1.31	.314
F	55	5.21	1.20	.361
G	53	5.81	0.86	.024

*P*-values from t-tests for binary categories and regression analyses for multiple categories.

ICHD, in-centre haemodialysis; HHD, home haemodialysis.

Key terms relating to the NPREM's development are defined in Table 8.

**Table 8:** Glossary of terms.

Term	Definition
Ceiling effect^a^	Denotes when participants’ responses fall towards the upper end of the response scale [46]
Codes/coding	Applying tags or labels to the data to help identify patterns. In qualitative methods, codes and coding are often initial steps in analysis [26]
Cognitive interviews	A qualitative method to assess if a measure (questionnaire) fulfils its intended purpose. Participants are usually from the target population [29]
Content validity	A term referring is the ‘degree to which elements of an assessment instrument are relevant to and representative of the targeted construct for a particular assessment purpose’ (p 238) [48]
Convergent validity	To assess if similar or theoretically related concepts are associated [23]
Dimensionality^b^	‘The latent structure of scale items and their underlying relationships’ (p.4) [23]. Scales can be unidimensional (one factor), bidimensional (two factors) or multi-dimensional (2+ factors)
Discriminant validity	Assessment of whether a scale's concept is different from another concept [23]
Expert panel	A mixed group of individuals with a variety of experiences and expertise related to needling, each having an equal voice in discussions
Face validity	The degree that the target population judge that a measure is appropriate to the construct and assessment objectives [23]
Item's content validity index (i-CVI)	A statistical method to assess interrater agreement which uses a proportional agreement [28]
Internal reliability/consistency	The degree to which the set of items in the scale co-vary, relative to their sum score, usually assessed with Cronbach's alpha [23]
Intra-class correlations (ICCs)	A statistical method used to describe how strongly measures from the same participant resemble each other over time
Known-groups validity	When a measure can differentiate between groups which we know a priori are likely to score differently [23]
Missing values (methods for handling)	Missing data presents a problem for analysis, and in general values are estimated for missing values to allow the effect of missing data to be evaluated (sensitivity analysis). There are a number of different methods for estimating what the missing values should be, with some methods using statistical modelling
Modified Kappa	A statistical method to determine interrater agreement. ‘The kappa statistic represents the proportion of agreement remaining after chance agreement is removed’ (p. 511) [28]
Order effect^c^	When the location of an item within the scale affects how the participant responds
Pearson's correlations	A statistical method to show associations between measures
Psychometric properties	A range of aspects related items within a measure and the measure itself which provide evidence to its usefulness and reliability
Reliability	The degree of consistency in the measure when it is repeated [23]
Root mean square error of approximation (RMSEA)	RMSEA is a statistic that tells how well a model fits the data. It measures the difference between what is expect to be seen in the data and what the model predicts, adjusted for the complexity of the model. The lower the RMSEA value, the better the model fits the data. General interpretations are <0.05 considered good, 0.05–0.08 acceptable, 0.08–0.1 marginal, >0.1 poor [40, 41]
Sensitivity analyses	Analyses conducted to evaluate whether the conclusions drawn from an analysis changes when missing data is accounted for. Under different assumptions and different estimating methods, missing data is replaced with a value. The analysis is rerun, and the outcome compared with the original analysis. Where replacement of missing data leads to a very different outcome, the main analysis is brought into question
Scale	A term used in survey methodology to denote an item or set of items relating to a core construct or theme. Other terms also commonly used: measure, questionnaire, survey, instrument, tool
Target population	The people with lived experience relating to the construct and who are the intended users of measure
Thematic analysis	A form of qualitative data analysis. The researchers identify themes (or reoccurring patterns or experiences) across the dataset. A thematic map is a visual representation of the themes
Think aloud	A technique used in cognitive interviewing. Participants complete the questionnaire while reading each item aloud and verbalizing their thoughts and response reasoning [29]
Validation/validated measure	An ongoing accumulation of evidence, following scale development guidance, which provides evidence for the accuracy of the measurement tool [23, 49]. The evidence should provide support showing that tool is capturing the properties of the underlying outcome of interest (validity), and that the tool can be used consistently in a particular setting or context (reliability).
Validity^d^	The extent to which a measure captures the construct it was designed to capture. There are various ways of testing validity, most commonly: content, construct (including convergent, discriminant, known group differentiation) and criterion [23]
Verbal probes	A technique in cognitive interviewing where the interviewer questions the participant about the item to gather further evidence [29]

a In the NPREM, this would be responses of 6 or 7, indicating very positive needling experience. In PREMs in particular which measure patient experience of care, it is not uncommon for participants to endorse the care they received [47].

b The results of the NPREM show needling experience to be unidimensional. This suggests that patient needling experience is one central concept.

c The cognitive interviews suggested a possible order effect regarding the painfulness of needling and overall experience items. In the pilot survey, these items were placed in different locations within the questionnaire to assess order effect.

d The choice of forms of validity can be difficult. For the NPREM, we utilized an assessment of convergent validity along with an assessment of divergent validity, as opposed to discriminant validity. There remains little research on patient experience formulated as a unitary measure, and little is known about the factors associated with these measures. Some definitions of discriminant validity refer to the ability for a scale to discriminate between factors, making this form of validity difficult to assess. We chose to focus on measures that were theoretically closer to the patient experience of cannulation (e.g. vascular access related quality of life) giving an assessment of convergent validity, and measures that were theoretically more distant (e.g. patient activation, health function) providing an assessment of divergent validity.

## DISCUSSION

Following a robust multi-phase development, NPREM(v1.0) provides a valid and reliable measure of patient experience of needling. It is patient-centred, developed with patients for patients, addressing important aspects of needling. It is a 28-item self-report questionnaire in which patients rate their current experience of needling across five themes and overall experience, providing a summary scale score with higher scores indicating positive experience.

This study is the first to develop a measure of patient-reported experience of needling for haemodialysis. Other measures focus on specific aspects of needling [16], whereas the NPREM(v1.0) captures the needling experience across areas of patients’ lives and care. The moderate correlation between the NPREM(v1.0) and the VASQoL suggests that although there is some overlap in concepts, overall patient experience of needling is a separate and unique concept. Likewise, the weak correlations between the NPREM(v1.0) and the EQ-5D-5L and PAM show that these too are distinct concepts and that the NPREM is not measuring health function or activation.

During item generation, we reviewed the wider literature and other measures to ensure that patient experience of needling was fully accounted. Our items reflected and extended concepts reported in the literature, many of which were conducted elsewhere in the world, offering some assurance that the NPREM captures the breadth of experience and may be applicable in wider haemodialysis populations.

One of the most significant findings in this study was that kidney centre was more strongly related to experience of needling than patient or clinical characteristics, as foreseen in patient experience of haemodialysis care in general [45]. Age was the only patient characteristic related to patient experiences of needling, with older people (>65 years) reporting more positively, also complementing results in the Kidney PREM [4].

Development and validation of the measure followed rigorous and widely accepted processes [23]. The Patient-Steering-Group collaborated in study design, set-up, delivery, analysis and dissemination, ensuring the measure maintained its patient-centred focus. Their involvement was complemented by a range of experts in the field, both clinical and methodological, as part of the expert panel and the Study-Steering-Group, ensuring clinical relevance and process rigour. A limitation was that kidney centre involvement was restricted to England. Further research should be conducted to confirm the measure's validity and applicability in other haemodialysis populations. The language of NPREM(v1.0) and its developmental predecessors were confined to English. In mitigation we included non-English speakers in interviews to identify potential differences in experience and in the surveys encouraged completion with assistance. Although our sample reflected the diversity of the UK patient population, it is possible that NPREM(v1.0) may not fully capture the experiences of non-English speakers.

Our aim was to develop a scale to collect evidence of patients’ experience of needling to inform clinical practice and quality improvement initiatives. The measure can be used to audit patient care at service-level or as part of clinical practice with individual patients. The NPREM may also provide the basis of national dataset benchmarking of patient experience of needling. There is also a potential use as an outcome measure in vascular access or needling studies.

Future research to facilitate implementation of the measure into routine clinical practice is required along with extension of its applicability to more diverse haemodialysis populations. Studies to understand the drivers of centre variation would also support improvement in needling practice.

The NPREM was robustly and rigorously developed to assess patient experience of needling for haemodialysis, with patients at the centre of the research. It is a self-report questionnaire with 28 items covering five themes of care. It offers a way to identify aspects of needling that are going well and those that could be improved at individual and service levels.

### Validated version of the NPREM(v1.0)

The NPREM(v1.0) is free to use, with the measure and scoring guidance provided (Supplementary data, SM13 and SM14). The copyright requests referencing this article when reporting use of the measure.

## Supplementary Material

sfaf029_Supplemental_File

## Data Availability

The authors confirm that the data supporting the findings of this study are available within the article and its supplementary materials.
