# Supplementary material for: Development and validation of a measure to assess patient experience of needling of arteriovenous fistulas or grafts for haemodialysis access: the NPREM
Source: Clin Kidney J. 2025 Jan 28;18(3):sfaf029. doi: 10.1093/ckj/sfaf029 (PMC11928787; doi:10.1093/ckj/sfaf029)
Supplement: sfaf029_Supplemental_File [file sfaf029_supplemental_file.docx]

# Supplementary material

## SM1: Additional supporting documents relating to this study

Additional supporting documents related to this study can be accessed here: <https://doi.org/10.17866/rd.salford.c.7366282>

- NPREM Study Protocol
- NPREM Patient and Public Involvement Methods
- NPREM Phase 1. Qualitative.Additional supporting quotations
- NPREM Phase 1. Item Generation.Expert panel overview and detailed actions

## SM2 NPREM Patient and Study Steering Groups & Expert Panel

The NPREM was supported throughout the programme of research by a Patient Steering Group and a Study Steering Group. An Expert Panel was convened to review the proposed items for the NPREM (Phase 1b). Summaries of each of the groups are provided below.

*NPREM Patient Steering Group*

A detailed description of the methods and how their involvement impacted the NPREM’s development are provided in the Supporting Documents (NPREM Patient and Public Involvement Methods, <https://doi.org/10.17866/rd.salford.26781187.v1>)

The NPREM Patient Steering Group consisted of people from the UK with lived experience of haemodialysis needling. Over the course of the NPREM’s development, 15 people with lived experience were involved in the study, leading to a co-produced NPREM. They reflected a range of needling experiences and backgrounds, which added depth and insight at each phase. The group met online via Zoom every 6-8 weeks throughout the study with an in-person two-day workshop at the end of the study to decide the final NPREM items, write sections of reports, and plan dissemination.

*NPREM Study Steering Group*

The NPREM Study Steering Group comprised 15 people with an interest in haemodialysis needling. This group was convened in November 2020 with its final meeting in June 2023. The group met quarterly to review the NPREM’s progress and troubleshoot any issues. The group included academics, methodologists and psychometricians with an interest in cannulation, scale development and/or patient experience; vascular access nurses (based within the UK and abroad); vascular access surgeon; nephrologists; haemodialysis nurses; representatives from kidney charity and industry; and people with lived experience of haemodialysis needling. The group was chaired by a member of the group independent of the research team.

*Expert Panel*

An expert panel was convened to assess whether the proposed items for the preliminary version of the NPREM (v0.1) were true to the qualitative findings and literature and to assess the relevance of any items where a consensus was not achieved within the research team. The group consisted of 10 people with an interest or professional experience in cannulation, psychometrics or patients experience, including: nurses (research, vascular access, haemodialysis), methodologist, psychometrician, nephrologist, policy lead, vascular access surgeon, and people with lived experience of haemodialysis needling. The expert panel met on two occasions (1 online workshop, 1 online voting) and was chaired by members of the research team. With the exception of 1 member of the expert panel who was also on the Study Steering Group, all other panel members were independent of the research.

## SM3 NPREM: Kidney centre characteristics

| *NHS Trusts* | *Phases* | *Fistula/Graft Usage** (High/Medium/Low) | *Number of units* |
| --- | --- | --- | --- |
| Barts Health NHS Trust | All | Medium | 5 |
| East and North Hertfordshire NHS Trust | All | Low | 5 |
| North Bristol NHS Trust | Phase 3 only | Low-Medium | 4 |
| Nottingham University Hospitals NHS Trust | Phases 1 & 2 | High | 5 |
| Portsmouth Hospitals NHS Trust | Phases 1 & 2 | Low-Medium | 10 |
| Northern Care Alliance NHS Foundation Trust | Phase 3 only | Medium | 5 |
| University Hospitals Birmingham NHS Foundation Trust | All | Medium-High | 8 |
| University Hospitals of Derby and Burton NHS Foundation Trust | All | High | 2 |
| * Fistula/Graft usage refers to the centres’ reported usage arteriovenous fistula or grafts at each centre, ranging from low to high usage, as reported in UK Renal Registry (2019).  Notes: Centres were chosen to reflect a balanced representation of low, middle and high AVG/AVF usage. In the UK, dialysis is provided by the NHS through kidney centres which look after patients across stages and treatments. | | | |

## SM4 NPREM Phase 1: Developing concepts and items - Key aspects of needling pertinent to patient experience

1. **The nature of needling**

- Needling is necessary for people on HD with a fistula/graft.
- It is often painful and can cause broader discomfort and anxiety.
- Needling experience varies from person to person and from session to session, with many factors affecting how it goes ‘on the day’.

1. **Health of the fistula or graft**

- The access is a lifeline, whose formation, maturation and care are fundamental to how needling goes.
- Some people are protective of their fistulas and do a lot to maintain its health.
- Concerns about longevity and alternatives can arise.
- There can be a keen awareness of the individual characteristics of fistulas and grafts and how these impact on needling.

1. **Needling process**

- There are many aspects of the needling process which can be done clinically adequately, but with negative impact (often pain) on the patient experience.
- This includes needle insertion, positioning and removal, with additional implications for sharp needles.
- Patients have different levels of awareness of these aspects, and of what makes an optimal experience for them.
- Taking time and recognising individual preferences are key.

1. **Past experiences**

- Positive experiences help to foster confidence in needling, can set expectations, and help people to tolerate negative experiences.
- Negative experiences stick out for people, are often associated with the needler, are more likely than positive experiences to set expectations, and can be a catalyst for change.
- Past experiences feed into people’s emotional responses to needling.

1. **Positive influences**

- Many patients have mechanisms they rely upon to help needling go well, which can serve to reduce worry and manage pain.
- Relaxation is recognised as being both mentally and physically important, some use specific techniques: routines, breathing, praying, distraction.
- Calling on support from family or friends is important, which may or may not include positive relationships in the unit.
- This theme does not include support from nursing staff.

1. **Who I am**

- Particularly complex theme.
- Draws on relatively stable psychological attributes which determine preferences around ownership of and engagement with needling as well as transitional aspects such as mood and things that impact mood on the dialysis day.
- Whilst acceptance of needling appears to be a positive influence, the inevitability of needling can be as much a reason to disengage and relinquish control as it can be to fully engage and want to take control.

1. **Working together**

- Needling is a two-person process between the needler and the needled, each bringing their own stable (preferences, outlook) and changeable (mood, stress) influences to every needling session.
- The mutual building of confidence and empathy makes for a more positive experience, as does communication.

1. **Contextual influences**

- Any of these are subject to changing and impacting needling on the day.
- These tend to be relatively stable and includes age, cultural background, other health issues or comorbidities, the renal unit, language barriers, and beliefs.

## SM5 NPREM Phase 1: Developing concepts and items - Results of item assessment and agreement by expert panel

| **#** | **Item** | **Item Content Validity** | | **Modified Kappa** | |
| --- | --- | --- | --- | --- | --- |
|  |  | **Index** | **Interpretation** | **Index** | **Interpretation** |
| 5 | Apart from pain, there were other things that affected my needling experience. | 0.88 | Retain | 0.87 | Excellent |
| 6 | Pain was the worst part of needling. | 0.38 | Exclude | 0.20 | Poor |
| 15 | I know my fistula/graft as well as I would like. | 0.38 | Exclude | 0.20 | Poor |
| 31 | I believe there are always opportunities to improve needling. | 0.50 | Exclude | 0.31 | Poor |
| 32 | I would like to know more about needling. | 0.88 | Retain | 0.87 | Excellent |
| 46 | I was comfortable with any of the nursing staff needling me. | 0.25 | Exclude | 0.16 | Poor |
| 55 | Support from others helped me feel positive about needling. | 0.75 | Revise | 0.72 | Good |
| Note: Values interpreted as i-CVI: >0.79 retain, 0.7-0.79 revise, <0.7 eliminate. Kappa: >0.75 excellent, 0.6-0.74 Good, 0.4-0.59 Fair, <0.4 Poor. | | | | | |

## SM6 NPREM Phase 2a: Cognitive interviews – From NPREM 0.1 to 0.1a


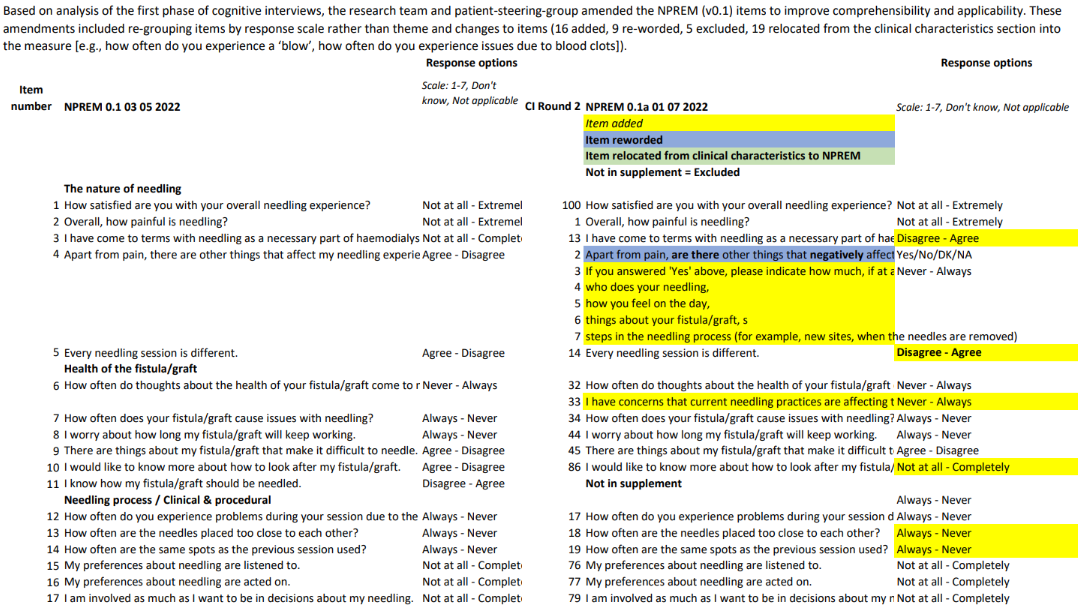


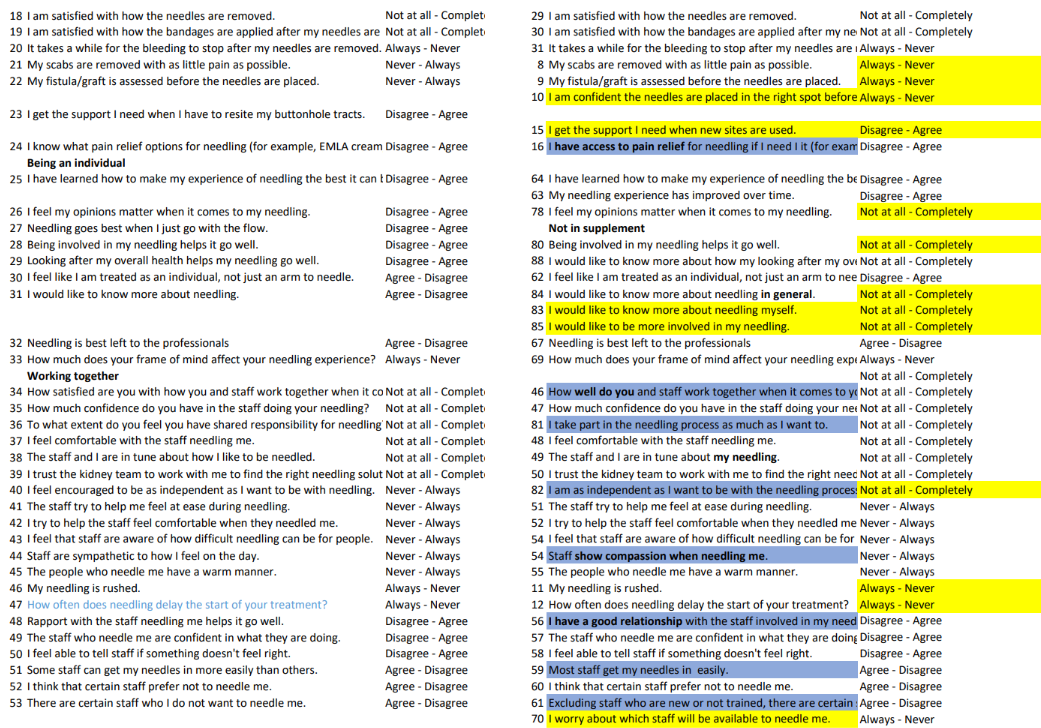


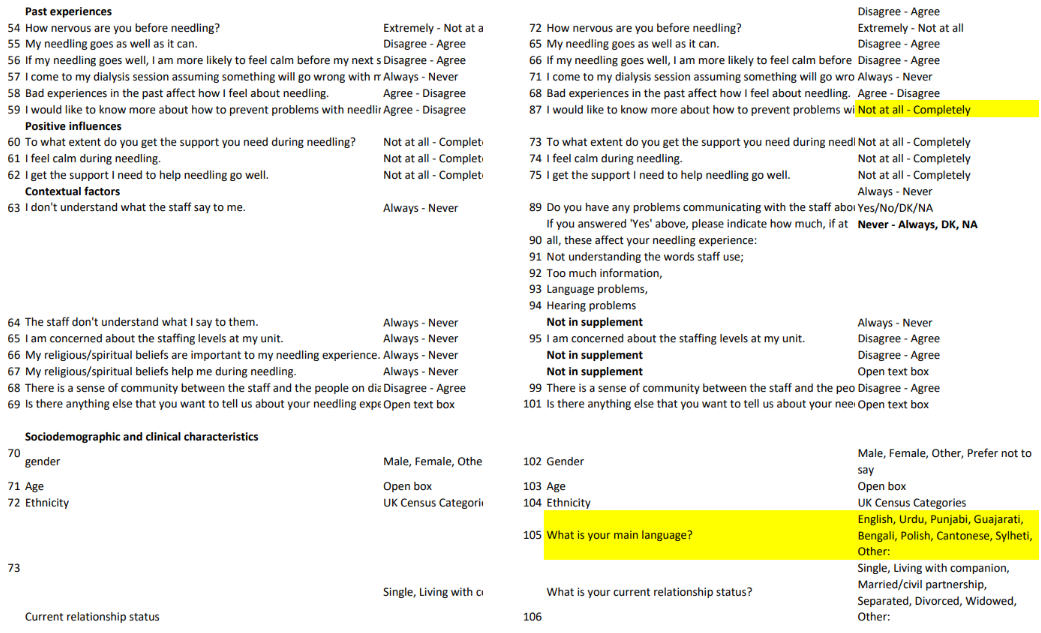


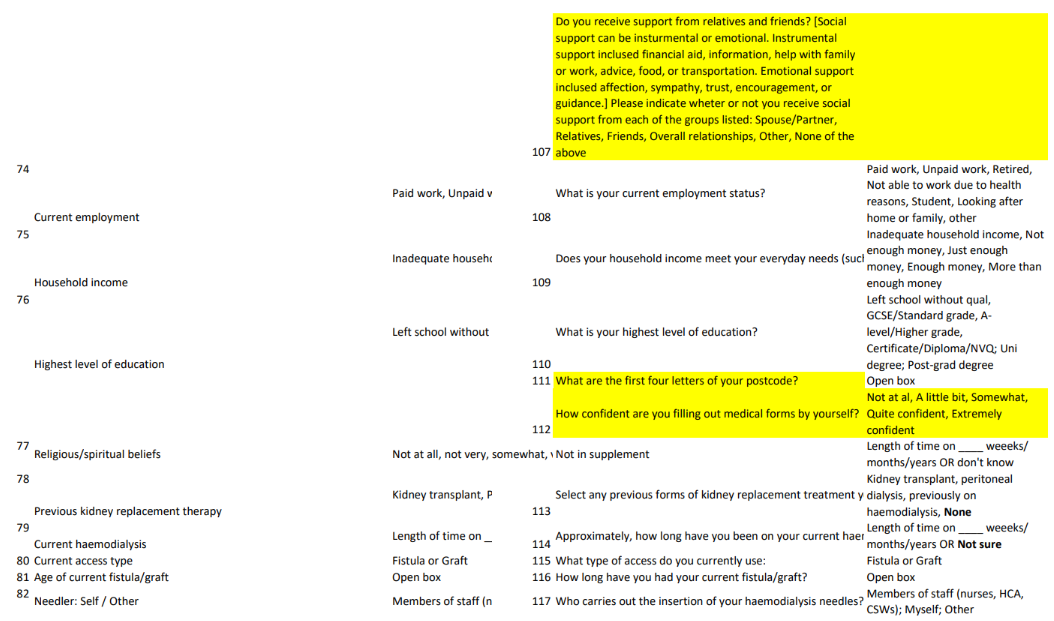


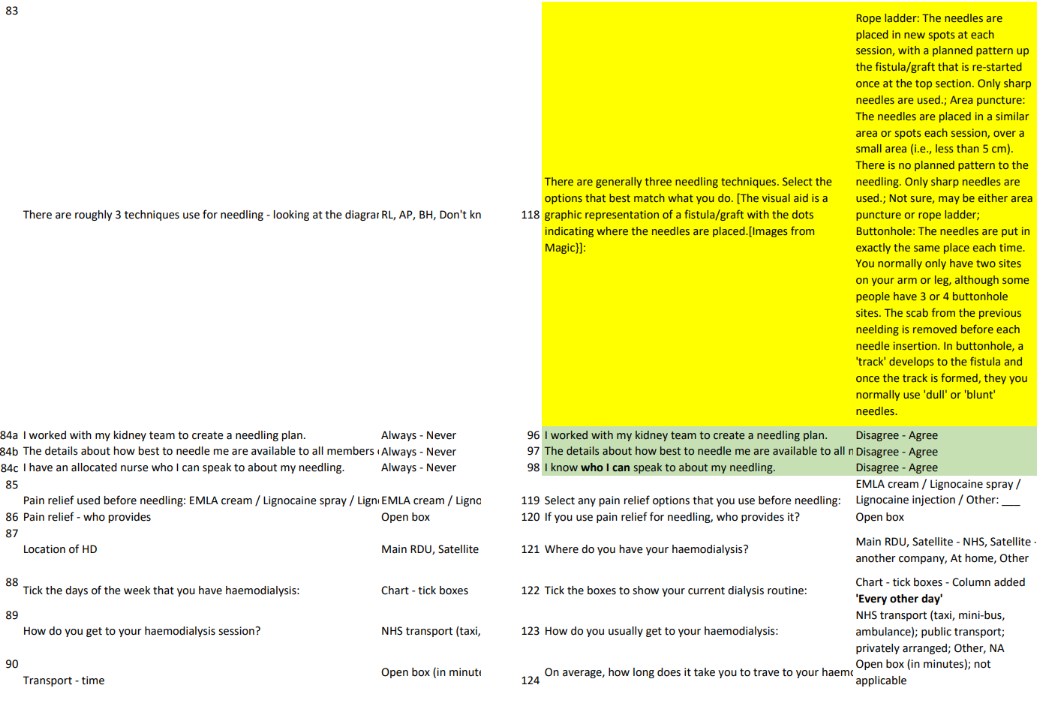


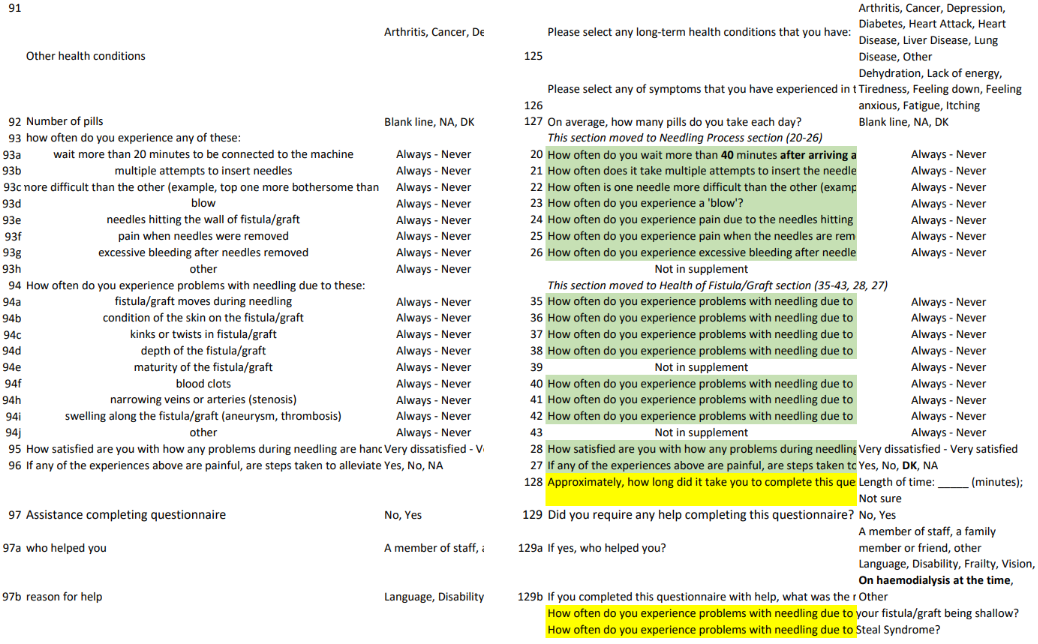


## SM7 NPREM Phase 2a: Cognitive interviews overview of item analysis and issues

| *Aspects of information processing* | *Definition* | *Example item, summary and action* |
| --- | --- | --- |
| *Comprehension* |  |  |
| Difficult item | Delay in comprehending question meaning and difficulty answering | Item: I know how my fistula/graft should be needled.  Summary: Difficult to draw any conclusions from the item - even if you know how you should be needled it may not be related to your experience. People who are less involved in the particulars of their needling do not see the relevance.  Action: Item excluded. |
| Hesitation | Excessive pausing or re-reading while comprehending the question | Item: How often does needling delay the start of your treatment?  Summary: Participants often hesitated and described factors outside of needling which delay them starting their session.  Action: Item excluded. |
| Incomprehension | The meaning of the question is not understood | Item: How often do you experience problems with needling due to the fistula/graft moving?  Summary: Some had no experience of rolling/moving fistulas or grafts so did not understand item. The wording 'during needling' made it difficult for one person to answer because the fistula/graft stays still during needling - it moves before needling.  Action: Item excluded. |
| Misinterpretation | Question not interpreted the way it was intended | Item: I am as independent as I want to be with the needling process.  Summary: Participants unsure how to interpret ‘independent’ and overall not well understood.  Action: Item excluded. |
| Multiple interpretations | There are two or more possible interpretations | Item: Do you have any problems communicating with the staff about your needling experience?  Summary: Participants interpreted this to be about the terminology staff used, the information they were given, being able to physically access staff to ask a question to not hearing what to communicate with them to their personal ability to understand information relating to needling.  Action: Tested a rephrased version of the item in the pilot: I have problems communicating with the staff about my needling. |
| Need for clarification | Participant needed more information to answer question | Item: My fistula/graft is assessed before the needles are placed.  Summary: 2 participants asked for more detail about what ‘assessed’ entailed. It aligned with their understanding.  Action: Item checked with patient steering group and included in pilot. |
| Semantic difficulties | The meaning of a word or phrase is not understood | Item: Rapport with the staff needling me helps it go well.  Summary: 1 participant did not know 'rapport' so other phrases tested (e.g., 'a good relationship'). Rapport deemed better than 'relationship'. stated safety more important than rapport and gist of item possibly covered in another item.  Action: Item rephrased. Items for pilot tested items with similar meaning but different wording (e.g., ‘feel at ease’) and added an item on ‘feeling safe’. |
| Wording | Issue with wording or phrasing of the question | Item: I am satisfied with how the bandages are applied after my needles are removed.  Summary: Participants discussed the phrasing at their centre (e.g., bandages, tapes).  Action: Rephrased using broader, more general term of ‘dressings’. |
| *Retrieval** |  |  |
| Lack of information | Participants did not have knowledge that could inform question | Item: My scabs are removed with as little pain as possible.  Summary: People who did not use buttonhole technique did understand this as they often had not heard of it.  Action: Added term ‘buttonhole’ to item to specify for particular group. |
| *Judgement* |  |  |
| Repetition | A question has the same meaning as a previous one | Item: I feel calm during needling.  Summary: Noted as similar to other items (‘nervous before needling’).  Action: Item excluded. |
| Relevance | The extent to which the question is relevant to their experience | Item: How often do you experience problems with needling due to Steal Syndrome?  Summary: Item not relevant to many people and those who did experience said it did not affect needling but haemodialysis and daily life in general.  Action: Item excluded. |
| Time frame | Refers to the reference point for answering the question | Item: How often are the needles placed too close to each other?  Summary: Participants reflected on events that happened in the distant past rather than current experience.  Action: Item excluded. |
| *Responding* |  |  |
| Change in time frame affects response** | If asked about the last needling session (rather than overall current experience), a different response selected. | Item: How much confidence do you have in the staff doing your needling?  Summary: Confidence may vary according to who puts you on/takes off. With the time frame ‘current experience’ means people averaged their experience.  Action: Adjusted phrasing to ‘How often do you have confidence…’ and response scale labels (Never – Always). Consensus to test item in pilot and evaluate psychometric properties. |
| Hesitation | Excessive pausing or hesitation | Item: How often does needling delay the start of your treatment?  Summary: Participants also hesitated when choosing a response, though often settled on a response that matched their reasoning. Hesitation may suggest that item is too complex or subtle for response scale.  Action: Item excluded. |
| Missed question | Question not answered, either intentionally or accidentally | Item: How often do you experience excessive bleeding after needles are removed?  Summary: Participant inadvertently skipped it. Review how items are presented as it may have been missed because grouped with items with very similar phrasing.  Action: Item excluded but item on length of time it takes to stop bleeding added to personal and clinical information section. |
| Response scale confusion | Difficulty when marking response on the scale | Item: How often do you experience problems with needling due to narrowing veins or arteries (stenosis)?  Summary: Participants chose answers that did not match reasoning or did not easily fit response scale.  Action: Item excluded. |
| Response scale scoring | Issues with the scoring of the response scale – includes issues with scale being flipped. | Item: How often do you experience problems during your session due to the placement of the needles?  Summary: Participants chose wrong response due to reversed response scale (response chosen was opposite of verbal reasoning).  Action: Rephrased to I experience problems due to the placement of the needles. |
| Response scale wording | Issues with wording of the response scale | Item: How satisfied are you with your overall needling experience?  Summary: Not at all – Extremely response label endpoints which do not align with item.  Action: Changed to Very dissatisfied – Very satisfied in pilot survey. |
| *Other* |  |  |
| Disparity | Disparity between understanding and reference points and response option chosen. | Item: I worry about how long my fistula/graft will keep working. Always – Never  Summary: Participants discussed how they always worry about their fistula/graft and chose responses that did not reflect their logic. Consider changing labels to capture the extent of their worry and reduce disparity.  Action: Item rephrased with new response labels: I worry about how long my fistula/graft will keep working. All the time – Not at all |
| Early HD** | At beginning of HD, may have answered differently | Item: How often does it take multiple attempts to insert the needles?  Summary: Participants often noted that in early days of needling it took several attempts. Overall viewed as an important item to signify needling issues.  Action: Item retained. |
| Formatting | Changes to format recommended, including item format (question vs statement) | Item: I have access to pain relief for needling if I need I it (for example, EMLA cream, lignocaine spray or injection).  Summary: Participants recommended simplifying information in brackets to ‘numbing cream, spray or injections’ and moving these to the end of item.  Action: Changes made to item and tested in pilot survey. |
| Limited applicability*** | Item may only apply to a sub-set of participants | Item: I try to help the staff feel comfortable when they needle me.  Summary: Item does not apply to people who needle themselves.  Action: Item excluded. Team reviewed retained items to ensure they are as broadly applicable as possible and apply to sub-groups. |  |
| New question*** | Participant suggests new question | Item: Do you have any problems communicating with the staff about your needling experience? [Yes, No, Don’t know, Not applicable; If you answered 'Yes' above, please indicate how much [Never - Always, DK, NA], if at all, these affect your needling experience: Not understanding the words staff use; Too much information, Language problems, Hearing problems.  Summary: Suggestions to add in other things that can prevent communication: Staff not available, Low mood - anxiety or depression.  Action: Item rephrased and simplified to ‘I have problems communicating with the staff about my needling’. |  |
| Order** | Change order of item in the questionnaire. | Item: Problems during needling are managed well.  Summary: Item placed in personal and clinical section; should be in main section of NPREM.  Action: Item moved to main section of NPREM for pilot survey. |  |
| Sociodemographic*** | Issue with question or response scale. | Item: What is your highest level of education?  Summary: Participants raised that many common sociodemographic questions may have little relevance to needling.  Action: Sociodemographic items reviewed and those with predicted associations with needling retained. |  |
| *Positive feedback* |  |  |  |
| Good question | Participant states that a question is good | Item: How well do you and staff work together when it comes to your needling?  Summary: Viewed as a good item by one participant but difficult to answer by another.  Action: Reviewed by team and consensus that other items capture this. Item excluded. |  |
| Important question** | Question highlighted as important | Item: My needling is rushed.  Summary: An important element of needling with people discussing what causes rushing and the outcome of rushed needling.  Action: Item retained. |  |
| Note. PSG=Patient-Steering-Group. Adapted from “The quality of life of people in chronic pain: Developing a pain and discomfort module for use with the WHOQOL,” by V. Mason, S. Skevington, and M. Osborn, 2008, Psychology & Health, 23, p. 139.  *Retrieval was added to the framework and adapted from *Cognitive Interviewing: A tool for improving questionnaire design*, by G. Willis, 2005, Sage Publications, pg.38.  ***Codes added by the research team.*  **** From Knapfl/PREM code book*  ***Bold***question numbers indicate deleted questions. | | |  |

## SM8 NPREM Phase 2b and 3: Development of NPREM items from pilot to evaluation phases

| **Pilot**  *V0.2*  *48 items* |  |  | **Evaluation**  *V0.3*  *35 items* |  |  | **Validated**  *V1.0*  *28 items* |  |  | **Actions** |
| --- | --- | --- | --- | --- | --- | --- | --- | --- | --- |
| *Item #* | *Theme (P1-P8)* | *Item* | *Item #* | *Theme (E1-E9)* | *Item* | *Item #* | *Theme (V1-5)* | *Item* |  |
| T1 | P1 | Overall, how painful is needling? | Q1 | E1 | No changes | Q1 | V5 | No change in wording | Regrouped to ‘My personal experience’ theme. Changed response scale labels to Not at all–Extremely and reverse scored. |
| T2 | P1 | How would you rate your overall needling experience? | Q30 | E1 | No changes | Q28 | Overall item | No changes | Based on pilot results on order effects, item placed at end of NPREM v0.3. |
| Q7 | P1 | I have come to terms with needling as a necessary part of haemodialysis. | - | - | - | - | - | - | Excluded on basis of psychometric properties in pilot |
| Q17 | P1 | I understand why my needling experience varies from session to session. | - | - | - | - | - | - | Excluded on basis of psychometric properties in pilot |
| Q43 | P1 | My needling experience varies greatly from session to session. | Q27 | E1 | No changes | Q22 | V5 | No change in wording | Regrouped to ‘My personal experience’ theme. |
| Q46 | P1 | How satisfied are you with your overall needling experience? | - | - | - | - | - | - | Excluded – Duplicate of Q1; Q1 better psychometric properties |
| Q20 | P2 | There are things about my fistula/graft that make it difficult to needle. | Q24 | E2 | No changes | Q20 | V1 | No changes | No additional comments |
| Q22 | P2 | I worry about how long my fistula/graft will keep working. | Q6 | E2 | No changes | Q5 | V1 | No changes | No additional comments |
| Q41 | P2 | I have concerns that current needling practices are harmful to my fistula/graft. | Q13 | E2 | No changes | Q12 | V1 | No changes | No additional comments |
| Q1 | P3 | Pain relief for needling is available to me if I need I it (for example, numbing cream, spray or injection). | S2 | E9 | No change in wording. | S2 | - | No changes to item wording but not part of NPREM scale | After pilot, item moved to ‘Service’ section to further assess item. After evaluation, item deemed not part of NPREM scale but important and to be collected alongside as part of sociodemographic section. Reworded to *I am aware that pain relief for needling is available to me (for example numbing cream, spray or injection)* with response scale: *Yes/No/ Don’t Know/Not Applicable* |
| Q5 | P3 | I am satisfied with how the needles are removed. | Q28 | E3 | I experience problems  when the needles are  removed. | Q23 | V2 | No changes | After pilot reworded to focus on experience rather than satisfaction. |
| Q11 | P3 | It takes multiple attempts to insert my needles. | Q25 | E3 | No changes | - | - | - | Excluded on basis of psychometric properties in evaluation. |
| Q12 | P3 | I am involved as much as I want to be in decisions about my needling. | Q9 | E7 | No changes | Q8 | V4 | No changes | After pilot became part of ‘Communication’ theme. |
| Q14 | P3 | Problems during needling are managed well. | Q12 | E6 | No changes | Q11 | V4 | No changes | After pilot became part of ‘Communication’ theme. |
| Q18 | P3 | My preferences about needling are acted on. | - | - | - | - | - | - | Excluded on basis of psychometric properties in pilot |
| Q23 | P3 | I get the support I need when new sites are used. | Q15 | E3 | I get the support I need  when new sites are  used (for example, when  new spots used along  the fistula/graft; for  people with buttonholes:  when new buttonholes  are formed). | Q27 | V2 | I get the support I need when new buttonhole sites are formed. | After pilot, reworded to clarify the meaning of item.  On basis of performance in evaluation, it was reworded and applicable only to buttonhole as this was an important aspect of needling for this sub-group and other items capture elements of needling that impact rope ladder and area puncture (pain, feeling safe, worry about needling practices). |
| Q24 | P3 | Information about how to needle me is available to the staff involved in my needling. | Q5 | E6 | No changes | S3 | - | Changes to wording | After evaluation, item deemed not part of NPREM scale but important and to be collected alongside as part of sociodemographic section. Reworded to as ‘*I have a needling care plan*’ with response scale: *Yes/No/ Don’t Know/Not Applicable* |
| Q25 | P3 | How often do you experience a 'blow'? | S4 | E9 | No change in wording | - | - | - | After pilot, item moved to ‘Service’ section to further assess item. After evaluation excluded due to continued poor psychometrics. |
| Q29 | P3 | The pain relief that I use works well. | S3 | E9 | No change in wording | Q25 | V2 | No change in wording | After pilot, item moved to ‘Service’ section to further assess item. In evaluation, moved into main NPREM items in the theme ‘Steps in Needling’ because it is an experience-based item important to a sub-set patients (high proportion but all other psychometrics good). |
| Q31 | P3 | I experience problems due to the placement of the needles. | Q2 | E3 | I experience problems  when the needles are  inserted. | Q2 | V2 | No changes | After pilot, item wording changed to reflect three stages in needling: insertion, positioning, and removal. |
| Q36 | P3 | My fistula/graft is assessed before the needles are placed. | Q21 | E3 | No changes | Q17 | V2 | No changes |  |
| Q40 | P3 | I am satisfied with how the dressings are applied after my needles are removed. | - | - | - | - | - |  | Excluded on basis of psychometric properties in pilot |
| Q45 | P3 | My buttonhole scabs are removed with as little pain as possible. | S5 | E9 | No change in wording | Q26 | V2 | No change in wording | After pilot, item moved to ‘Service’ section to further assess item.  On the basis of evaluation, this item was retained as it is an important aspect of needling for a sub-set of patients and included as part of the main NPREM in the theme ‘Steps in Needling’. |
| - | - | - | Q17 | E3 | I experience problems  due to the positioning of  the needles once they  are inserted. | Q15 | V2 | No changes | Added after pilot survey to capture a key element of needling. |
| Q2 | P4 | My needling is rushed. | Q7 | E4 | No changes | Q6 | V3 | No changes |  |
| Q10 | P4 | I trust the kidney team when it comes to my needling. | Q3 | E4 | No changes | Q3 | V3 | I trust the dialysis team when it comes to my needling. | After evaluation phase, minor rephrasing ‘kidney team’ changed to ‘dialysis team’ on basis of feedback from PSG. |
| Q13 | P4 | I feel the staff needling me show empathy. | Q8 | E4 | No changes | Q7 | V3 | I feel the dialysis team needling me show empathy. | After evaluation phase, minor rephrasing ‘staff’ changed to ‘dialysis team’ on basis of feedback from PSG. |
| Q16 | P4 | I feel comfortable with the staff who needle me. | - | - | - | - | - | - | Excluded on basis of psychometric properties in pilot |
| Q19 | P4 | The staff try to help me feel at ease during needling. | Q23 | E4 | No changes | Q19 | V3 | The dialysis team put me feel at ease during needling. | After evaluation phase, minor rephrasing ‘staff’ changed to ‘dialysis team’ and modified phrase ‘try to help me’ changed to ‘put’ on basis of feedback from PSG. |
| Q28 | P4 | How often do you have confidence in the staff who needle you? | - | - | - | - | - | - | Excluded on basis of psychometric properties in pilot |
| Q34 | P4 | I worry about which staff will be available to needle me. | Q16 | E4 | No changes | Q14 | V3 | I worry about who will be available to needle me. | After evaluation phase, minor rephrasing ‘which staff’ changed to ‘who’ on basis of feedback from PSG. |
| Q35 | P4 | I know who I can speak to about my needling. | Q18 | E5 | No changes | - | - | - | Excluded on basis of psychometric properties in evaluation |
| Q37 | P4 | I feel rapport with the staff needling me. | - | - | - | - | - | - | Excluded on basis of psychometric properties in pilot |
| Q38 | P4 | My needling is done in a way that makes me feel safe. | Q11 | E4 | No changes | Q10 | V3 | No changes |  |
| Q39 | P4 | My kidney team works with me to solve problems related to my needling. | Q20 | E4 | No changes | - | - | - | Excluded on basis of psychometric properties in evaluation |
| Q42 | P4 | I feel able to tell staff if something doesn't feel right. | Q26 | E5 | No changes | Q21 | V4 | I feel able to tell the dialysis team if something doesn't feel right. | After pilot became part of ‘Communication’ theme.  After evaluation phase, minor rephrasing ‘staff’ changed to ‘the dialysis team’ on basis of feedback from PSG. |
| Q6 | P5 | Needling is best left to the professionals. | - | - | - | - | - | - | Excluded on basis of psychometric properties in pilot |
| Q21 | P5 | My frame of mind affects my needling experience. | Q10 | E6 | No changes | Q9 | V5 | No changes |  |
| Q30 | P5 | My opinions about needling are taken seriously by the staff. | Q14 | E7 | No changes | Q13 | V4 | My opinions about needling are taken seriously by the dialysis team. | After pilot became part of ‘Communication’ theme.  After evaluation phase, minor rephrasing ‘staff’ changed to ‘dialysis team’ on basis of feedback from PSG. |
| Q32 | P5 | I take part in the needling process as much as I want to. | - | - | - | - | - | - | Excluded on basis of psychometric properties in pilot |
| Q33 | P5 | My needling experience has improved over time. | Q4 | E6 | No changes | Q4 | V5 | No changes |  |
| Q44 | P5 | I feel like I am treated as a person, not just an arm to needle. | - | - | - | - | - | - | Excluded on basis of psychometric properties in pilot |
| Q27 | P6 | I get the support I need to help needling go well. | - | - | - | - | - | - | Excluded on basis of psychometric properties in pilot |
| Q4 | P7 | Previous bad experiences of needling still affect how I feel about my needling. | Q29 | E8 | No changes | Q24 | V5 | No changes |  |
| Q9 | P7 | My needling goes as well as it can. | - | - | - | - | - | - | Excluded on basis of psychometric properties in pilot |
| Q26 | P7 | I am nervous before needling. | Q19 | E8 | No changes | Q16 | V5 | No changes |  |
| Q3 | P8 | I have problems communicating with the staff about my needling. | Q22 | E5 | No changes | Q18 | V4 | I have problems communicating with the dialysis team about my needling. | After pilot became part of ‘Communication’ theme.  After evaluation phase, minor rephrasing ‘staff’ changed to ‘dialysis team’ on basis of feedback from PSG. |
| Q8 | P8 | I am concerned about the staffing levels at my unit. | S1 | E9 | No change in wording | S1 | - | No change in wording | After pilot, item moved to ‘Service’ section to further assess item. After evaluation, item deemed not part of NPREM scale but important and to be collected alongside as part of sociodemographic section. |
| Q15 | P8 | There is a sense of community between the staff and the people on dialysis. | - | - | - | - | - | - | Excluded on basis of psychometric properties in pilot |
| Notes: PSG=Patient Steering Group, T1=Test1, T2=Test2. The pilot themes reflect themes developed in interviews and carried over to cognitive interviews. Pilot themes: P1:Nature of needling, P2: Health of the fistula/graft, P3: Needling process, P4: Working together, P5: Being an individual, P6: Positive influences, P7: Past experiences, P8: Context. Evaluation themes: E1: Overall, E2: Health of the fistula/graft, E3: Needling process, E4: Working together, E5: Communication, E6: Being an individual, E7: Involvement, E8: Past experiences, E9: Service. Validated themes: V1 My Fistula/Graft and Needling, Steps in Needling, Working Together, Communicating with the Team, My Personal Experience. | | | | | | | | | |

## SM9 NPREM Phase 2b: Piloting the measure - Item response profile and internal consistency of the NPREM v0.2 (n=183)

| **Item** | **Sign** | **Item response profile** | | | | | | | | **Item fit statistics** | | | | | | |  |
| --- | --- | --- | --- | --- | --- | --- | --- | --- | --- | --- | --- | --- | --- | --- | --- | --- | --- |
|  |  | *N* | *Mean (SD)* | *Median* | *N/A* | *Don't know* | *Missing (%)* | *1-3 scores* | *7 scores* | | *Factor Loading* | *Uniqueness* | *Item-test correlation* | *Item-rest correlation* | *Average inter-item covariance* | *Alpha if removed* | |
| T1 | - | 178 | 4.88 (1.90) | 5 | 0 | 0 | 2 (1.1%) | 42 (23.6%) | 44 (24.7%) | | 0.456 | 0.459 | 0.509 | 0.473 | 0.290 | 0.951 | |
| T2 | + | 179 | 6.05 (1.36) | 7 | 0 | 1 | 2 (1.1%) | 11 (6.1%) | 99 (55.3%) | | ¥ | ¥ | 0.679 | 0.655 | 0.286 | 0.949 | |
| Q1 | + | 104 | 5.12 (2.62) | 7 | 57 | 18 | 3 (1.6%) | 29 (27.9%) | 63 (60.6%) | | 0.132 | 0.721 | -0.113 | -0.156 | 0.299 | 0.953 | |
| Q2 | - | 173 | 6.41 (1.30) | 7 | 5 | 1 | 3 (1.6%) | 11 (6.4%) | 130 (75.1%) | | 0.621 | 0.322 | 0.644 | 0.617 | 0.286 | 0.950 | |
| Q3 | - | 169 | 6.51 (1.36) | 7 | 10 | 0 | 2 (1.1%) | 10 (5.9%) | 142 (84.0%) | | 0.415 | 0.436 | 0.464 | 0.430 | 0.291 | 0.951 | |
| Q4 | - | 162 | 5.62 (1.86) | 7 | 16 | 2 | 2 (1.1%) | 24 (14.8%) | 83 (51.2%) | | 0.611 | 0.372 | 0.639 | 0.614 | 0.287 | 0.950 | |
| Q5 | + | 181 | 6.60 (1.10) | 7 | 0 | 0 | 2 (1.1%) | 5 (2.8%) | 149 (82.3%) | | 0.563 | 0.337 | 0.552 | 0.494 | 0.288 | 0.950 | |
| Q6 | - | 176 | 2.65 (2.52) | 1 | 2 | 0 | 3 (1.6%) | 125 (71.0%) | 38 (21.6%) | | -0.050 | 0.663 | -0.009 | -0.052 | 0.304 | 0.954 | |
| Q7 | + | 176 | 6.63 (1.19) | 7 | 2 | 1 | 3 (1.6%) | 8 (4.5%) | 153 (86.9%) | | 0.497 | 0.421 | 0.514 | 0.483 | 0.290 | 0.951 | |
| Q8 | - | 166 | 4.81 (2.24) | 6 | 4 | 7 | 3 (1.6%) | 48 (28.9%) | 60 (36.1%) | | 0.390 | 0.591 | 0.419 | 0.384 | 0.292 | 0.951 | |
| Q9 | + | 177 | 6.42 (1.18) | 7 | 2 | 0 | 3 (1.6%) | 7 (4.0%) | 126 (71.2%) | | 0.584 | 0.431 | 0.619 | 0.591 | 0.288 | 0.950 | |
| Q10 | + | 177 | 6.56 (1.08) | 7 | 1 | 2 | 2 (1.1%) | 8 (4.5%) | 141 (79.7%) | | 0.625 | 0.272 | 0.623 | 0.594 | 0.286 | 0.950 | |
| Q11 | - | 176 | 5.97 (1.26) | 6 | 1 | 1 | 2 (1.1%) | 11 (6.3%) | 77 (43.8%) | | 0.358 | 0.477 | 0.386 | 0.350 | 0.293 | 0.951 | |
| Q12 | + | 175 | 6.32 (1.57) | 7 | 2 | 1 | 3 (1.6%) | 15 (8.6%) | 133 (76.0%) | | 0.464 | 0.468 | 0.494 | 0.460 | 0.290 | 0.951 | |
| Q13 | + | 171 | 6.43 (1.36) | 7 | 6 | 1 | 5 (2.7%) | 11 (6.4%) | 132 (77.2%) | | 0.586 | 0.392 | 0.645 | 0.595 | 0.287 | 0.950 | |
| Q14 | + | 175 | 6.62 (0.88) | 7 | 4 | 0 | 3 (1.6%) | 5 (2.9%) | 137 (78.3%) | | 0.740 | 0.274 | 0.746 | 0.727 | 0.284 | 0.949 | |
| Q15 | + | 178 | 6.58 (1.02) | 7 | 0 | 1 | 3 (1.6%) | 4 (2.2%) | 141 (79.2%) | | 0.671 | 0.268 | 0.662 | 0.638 | 0.286 | 0.950 | |
| Q16 | + | 173 | 6.53 (0.97) | 7 | 5 | 2 | 3 (1.6%) | 3 (1.7%) | 129 (74.6%) | | 0.760 | 0.167 | 0.757 | 0.738 | 0.284 | 0.949 | |
| Q17 | + | 172 | 6.10 (1.61) | 7 | 4 | 4 | 2 (1.1%) | 14 (8.1%) | 109 (63.4%) | | 0.248 | 0.594 | 0.276 | 0.235 | 0.296 | 0.952 | |
| Q18 | + | 153 | 6.36 (1.41) | 7 | 14 | 12 | 4 (2.2%) | 9 (5.9%) | 111 (72.5%) | | 0.612 | 0.350 | 0.642 | 0.616 | 0.286 | 0.950 | |
| Q19 | + | 175 | 6.55 (1.16) | 7 | 4 | 0 | 2 (1.1%) | 7 (4.0%) | 140 (80.0%) | | 0.699 | 0.177 | 0.688 | 0.664 | 0.285 | 0.949 | |
| Q20 | - | 155 | 5.68 (1.83) | 7 | 11 | 10 | 5 (2.7%) | 23 (14.8%) | 82 (52.9%) | | 0.306 | 0.460 | 0.342 | 0.305 | 0.294 | 0.951 | |
| Q21 | + | 166 | 5.82 (1.83) | 7 | 5 | 3 | 7 (3.8%) | 22 (13.3%) | 97 (58.4%) | | 0.523 | 0.351 | 0.539 | 0.509 | 0.289 | 0.950 | |
| Q22 | - | 173 | 5.03 (2.25) | 6 | 0 | 4 | 4 (2.2%) | 47 (27.2%) | 79 (45.7%) | | 0.356 | 0.396 | 0.392 | 0.355 | 0.293 | 0.951 | |
| Q23 | + | 128 | 6.36 (1.38) | 7 | 40 | 11 | 4 (2.2%) | 9 (7.0%) | 93 (72.7%) | | 0.513 | 0.404 | 0.554 | 0.527 | 0.288 | 0.950 | |
| Q24 | + | 142 | 6.20 (1.59) | 7 | 4 | 32 | 4 (2.2%) | 13 (9.2%) | 95 (66.9%) | | 0.559 | 0.422 | 0.616 | 0.589 | 0.287 | 0.950 | |
| Q25 | + | 154 | 5.75 (1.28) | 6 | 6 | 16 | 4 (2.2%) | 11 (7.1%) | 45 (29.2%) | | 0.177 | 0.747 | -0.136 | -0.177 | 0.305 | 0.954 | |
| Q26 | - | 175 | 5.27 (2.22) | 6 | 0 | 1 | 6 (3.3%) | 42 (24.0%) | 84 (48.0%) | | 0.521 | 0.301 | 0.529 | 0.498 | 0.290 | 0.950 | |
| Q27 | + | 174 | 6.40 (1.30) | 7 | 3 | 2 | 3 (1.6%) | 10 (5.7%) | 127 (73.0%) | | 0.725 | 0.227 | 0.718 | 0.695 | 0.284 | 0.949 | |
| Q28 | + | 175 | 6.43 (1.01) | 7 | 4 | 0 | 3 (1.6%) | 4 (2.3%) | 116 (66.3%) | | 0.655 | 0.302 | 0.649 | 0.624 | 0.286 | 0.950 | |
| Q29 | + | 54 | 5.52 (1.91) | 6 | 116 | 8 | 3 (1.6%) | 8 (14.8%) | 25 (46.3%) | | 0.258 | 0.649 | 0.473 | 0.441 | 0.289 | 0.950 | |
| Q30 | + | 152 | 6.49 (1.24) | 7 | 14 | 15 | 2 (1.1%) | 8 (5.3%) | 116 (76.3%) | | 0.672 | 0.284 | 0.706 | 0.684 | 0.285 | 0.949 | |
| Q31 | - | 171 | 5.98 (1.28) | 6 | 4 | 6 | 2 (1.1%) | 10 (5.8%) | 82 (48.0%) | | 0.468 | 0.348 | 0.498 | 0.466 | 0.290 | 0.951 | |
| Q32 | + | 137 | 5.70 (2.07) | 7 | 32 | 11 | 2 (1.1%) | 21 (15.3%) | 82 (59.9%) | | 0.495 | 0.469 | 0.559 | 0.531 | 0.289 | 0.950 | |
| Q33 | + | 172 | 6.01 (1.52) | 7 | 4 | 3 | 2 (1.1%) | 11 (6.4%) | 101 (58.7%) | | 0.573 | 0.375 | 0.597 | 0.568 | 0.288 | 0.950 | |
| Q34 | - | 171 | 5.47 (2.10) | 7 | 6 | 1 | 4 (2.2%) | 36 (21.1%) | 91 (53.2%) | | 0.729 | 0.171 | 0.734 | 0.714 | 0.284 | 0.949 | |
| Q35 | + | 168 | 6.51 (1.25) | 7 | 5 | 5 | 4 (2.2%) | 10 (6.0%) | 135 (80.4%) | | 0.688 | 0.282 | 0.704 | 0.682 | 0.285 | 0.949 | |
| Q36 | + | 162 | 6.12 (1.77) | 7 | 2 | 13 | 5 (2.7%) | 18 (11.1%) | 118 (72.8%) | | 0.508 | 0.429 | 0.538 | 0.505 | 0.289 | 0.950 | |
| Q37 | + | 171 | 6.57 (1.02) | 7 | 5 | 1 | 5 (2.7%) | 5 (2.9%) | 133 (77.8%) | | 0.647 | 0.204 | 0.647 | 0.621 | 0.286 | 0.950 | |
| Q38 | + | 177 | 6.63 (0.90) | 7 | 1 | 0 | 4 (2.2%) | 2 (1.1%) | 141 (79.7%) | | 0.824 | 0.162 | 0.803 | 0.788 | 0.282 | 0.949 | |
| Q39 | + | 160 | 6.42 (1.34) | 7 | 11 | 8 | 4 (2.2%) | 10 (6.3%) | 119 (74.4%) | | 0.689 | 0.269 | 0.721 | 0.698 | 0.285 | 0.949 | |
| Q40 | + | 176 | 6.64 (0.89) | 7 | 1 | 1 | 4 (2.2%) | 3 (1.7%) | 139 (79.0%) | | 0.543 | 0.351 | 0.536 | 0.506 | 0.289 | 0.950 | |
| Q41 | - | 162 | 6.33 (1.37) | 7 | 4 | 13 | 2 (1.1%) | 11 (6.8%) | 114 (70.4%) | | 0.493 | 0.425 | 0.516 | 0.485 | 0.290 | 0.950 | |
| Q42 | + | 180 | 6.76 (0.94) | 7 | 0 | 0 | 2 (1.1%) | 5 (2.8%) | 164 (91.1%) | | 0.397 | 0.447 | 0.430 | 0.395 | 0.293 | 0.951 | |
| Q43 | + | 179 | 5.26 (2.14) | 6 | 0 | 1 | 2 (1.1%) | 41 (22.9%) | 76 (42.5%) | | 0.411 | 0.550 | 0.447 | 0.411 | 0.292 | 0.951 | |
| Q44 | + | 179 | 6.68 (0.94) | 7 | 2 | 0 | 1 (0.5%) | 5 (2.8%) | 150 (83.8%) | | 0.620 | 0.327 | 0.621 | 0.593 | 0.287 | 0.950 | |
| Q45 | + | 67 | 5.97 (1.86) | 7 | 103 | 8 | 4 (2.2%) | 9 (13.4%) | 43 (64.2%) | | 0.369 | 0.587 | 0.559 | 0.533 | 0.288 | 0.950 | |
| Q46 |  | 180 | 6.44 (1.08) | 7 | 0 | 1 | 1 (0.5%) | 6 (3.3%) | 124 (68.9%) | | ¥ | ¥ | 0.821 | 0.806 | 0.282 | 0.949 | |
| Note. Responses captured using a 1 to 7 Likert scale with labels at endpoints with ‘Don’t Know’ and ‘Not Applicable’ options. Higher scores reflect positive needling experience. | | | | | | | | | | | | | | | | |  |

## SM10 NPREM Phase 2b: Piloting the measure - Eigenvalues of the first six factors for the NPREM preliminary version 0.2 (exploratory factor analysis, n=178)

| **Factor** | **Eigenvalue** | **Proportion of variance** | **Cumulative variance** |
| --- | --- | --- | --- |
| Factor 1 | 13.684^a)^ | 0.534 | 0.534 |
| Factor 2 | 2.134 | 0.083 | 0.618 |
| Factor 3 | 1.695 | 0.066 | 0.684 |
| Factor 4 | 1.273 | 0.050 | 0.734 |
| Factor 5 | 1.025 | 0.040 | 0.774 |
| Factor 6 | 0.951 | 0.037 | 0.811 |

1. *The high Eigenvalue for factor 1 suggests a single factor model consisting of all questions.*

## SM11 NPREM Phase 2b: Piloting the measure - NPREM v0.2 scree plot ( exploratory factor analysis, n=178)


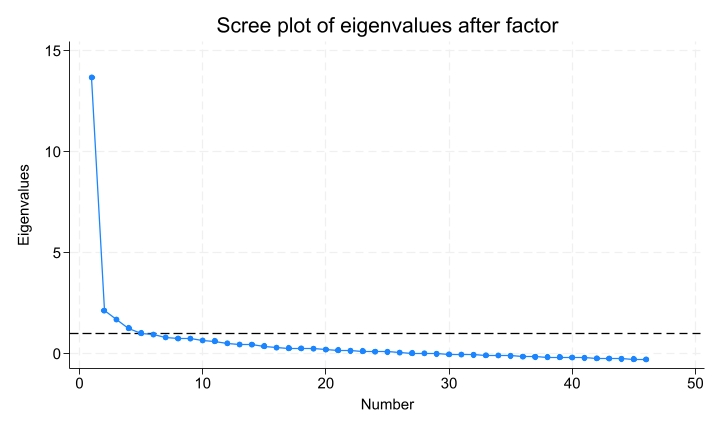


## SM12 NPREM Phase 2b: Piloting the measure – Outcomes of order effect assessment

|  | **T1: How painful overall (n=178)** | | | **T2: Overall experience (n=179)** | | |
| --- | --- | --- | --- | --- | --- | --- |
|  | **A** | **B** | **C** | **A** | **B** | **C** |
| 1 | 4 (6.7%) | 4 (6.6%) | 7 (12.3%) | 0 (0.0%) | 0 (0.0%) | 1 (1.8%) |
| 2 | 3 (5.0%) | 4 (6.6%) | 4 (7.0%) | 0 (0.0%) | 0 (0.0%) | 4 (7.0%) |
| 3 | 5 (8.3%) | 7 (11.5%) | 4 (7.0%) | 2 (3.3%) | 3 (4.9%) | 1 (1.8%) |
| 4 | 8 (13.3%) | 11 (18.0%) | 5 (8.8%) | 6 (10.0%) | 6 (9.8%) | 6 (10.5%) |
| 5 | 15 (25.0%) | 6 (9.8%) | 8 (14.0%) | 2 (3.3%) | 4 (6.6%) | 9 (15.8%) |
| 6 | 12 (20.0%) | 12 (19.7%) | 15 (26.3%) | 10 (16.7%) | 13 (21.3%) | 13 (22.8%) |
| 7 | 13 (21.7%) | 17 (27.9%) | 14 (24.6%) | 41 (68.3%) | 36 (59.0%) | 22 (38.6%) |
| **Mean (SD)** | 4.92 (1.76) | 4.89 (1.91) | 4.82 (2.06) | 6.34 (1.12) | 6.18 (1.21) | 5.59 (1.62) |
| **t-test p-values** | **A vs B** | **A vs C** | **B vs C** | **A vs B** | **A vs C** | **B vs C** |
|  | 0.9251 | 0.7951 | 0.8684 | 0.4323 | 0.004 | 0.0261 |
| **Fisher's Exact test** | 0.652 | | | 0.036 | | |

NPREM A: T1 at beginning, T2 after Q10

NPREM B: T1 and T2 after Q10

NPREM C: T2 at beginning, T1 at end (before overall satisfaction question)

## SM13 NPREM v1.0

**Needling Patient Reported Experience Measure**

**NPREM**

**Version 1.0**

**About the questionnaire:**
This questionnaire will ask you about a range of topics related to your current needling experience. Items are presented in a random order.

**Instructions**

- You should **circle the number** that best matches your **current** experience. Every item has the option of numbers 1-7. Please note, the response labels change from item to item. It is important that you read each item and your response options carefully.
- You may circle *Don’t know* or *Not applicable* if one of these best matches your experience.
- If you are unsure which response to circle, please choose the one that feels most appropriate. This is often your first instinct.

*[INSERT ANY ADDITIONAL INSTRUCTIONS]*

Please think about your **current experience of needling**

*©*The NPREM can be freely used with reference to Moore, C. et al. 2024. Development and validation of a measure to assess patient experience of needling of arteriovenous fistulas or grafts for haemodialysis access: The NPREM. Under-review. *Clinical Kidney Journal.* Any modification will compromise validity and must be clearly stated in reports.

**About your needling experience**

Needling is the placement of haemodialysis needles into your fistula or graft.

|  |  | *Not at all painful* | |  |  |  | *Extremely*  *painful* | | |  |
| --- | --- | --- | --- | --- | --- | --- | --- | --- | --- | --- |
| Q  1 | Overall, how painful is needling? | 1 | 2 | 3 | 4 | 5 | 6 | 7 | Don’t know | Not applicable |

There are many other aspects that make up your needling experience. The following questions address these.

|  |  | *Always* | |  |  |  | *Never* | | |  |
| --- | --- | --- | --- | --- | --- | --- | --- | --- | --- | --- |
| Q  2 | I experience problems when the needles are inserted. | 1 | 2 | 3 | 4 | 5 | 6 | 7 | Don’t know | Not applicable |

|  |  | *Not at all* | |  |  |  | *Completely* | | |  |
| --- | --- | --- | --- | --- | --- | --- | --- | --- | --- | --- |
| Q 3 | I trust the dialysis team when it comes to my needling. | 1 | 2 | 3 | 4 | 5 | 6 | 7 | Don’t know | Not applicable |

|  |  | *Strongly disagree* | |  |  |  | *Strongly*  *agree* | | |  |
| --- | --- | --- | --- | --- | --- | --- | --- | --- | --- | --- |
| Q  4 | My needling experience has improved over time. | 1 | 2 | 3 | 4 | 5 | 6 | 7 | Don’t know | Not applicable |

|  |  | *All the time* | |  |  |  | *Not at all* | | |  |
| --- | --- | --- | --- | --- | --- | --- | --- | --- | --- | --- |
| Q 5 | I worry about how long my fistula/graft will keep working. | 1 | 2 | 3 | 4 | 5 | 6 | 7 | Don’t know | Not applicable |

|  |  | *Always* | |  |  |  | *Never* | | |  |
| --- | --- | --- | --- | --- | --- | --- | --- | --- | --- | --- |
| Q 6 | My needling is rushed. | 1 | 2 | 3 | 4 | 5 | 6 | 7 | Don’t know | Not applicable |

|  |  | *Never* | |  |  |  | *Always* | | |  |
| --- | --- | --- | --- | --- | --- | --- | --- | --- | --- | --- |
| Q 7 | I feel that the dialysis team needling me show empathy. | 1 | 2 | 3 | 4 | 5 | 6 | 7 | Don’t know | Not applicable |

|  |  | *Not at all* | |  |  |  | *Completely* | | |  |
| --- | --- | --- | --- | --- | --- | --- | --- | --- | --- | --- |
| Q 8 | I am involved as much as I want to be in decisions about my needling. | 1 | 2 | 3 | 4 | 5 | 6 | 7 | Don’t know | Not applicable |

|  |  | *Always* | |  |  |  | *Never* | | |  |
| --- | --- | --- | --- | --- | --- | --- | --- | --- | --- | --- |
| Q 9 | My frame of mind affects my needling experience. | 1 | 2 | 3 | 4 | 5 | 6 | 7 | Don’t know | Not applicable |

|  |  | *Never* | |  |  |  | *Always* | | |  |
| --- | --- | --- | --- | --- | --- | --- | --- | --- | --- | --- |
| Q  10 | My needling is done in a way that makes me feel safe. | 1 | 2 | 3 | 4 | 5 | 6 | 7 | Don’t know | Not applicable |

|  |  | *Never* | |  |  |  | *Always* | | |  |
| --- | --- | --- | --- | --- | --- | --- | --- | --- | --- | --- |
| Q 11 | Problems during needling are managed well. | 1 | 2 | 3 | 4 | 5 | 6 | 7 | Don’t know | Not applicable |

|  |  | *Strongly agree* | |  |  |  | *Strongly*  *disagree* | | |  |
| --- | --- | --- | --- | --- | --- | --- | --- | --- | --- | --- |
| Q  12 | I have concerns that current needling practices are harmful to my fistula/graft. | 1 | 2 | 3 | 4 | 5 | 6 | 7 | Don’t know | Not applicable |

|  |  | *Strongly disagree* | |  |  |  | *Strongly*  *agree* | | |  |
| --- | --- | --- | --- | --- | --- | --- | --- | --- | --- | --- |
| Q  13 | My opinions about needling are taken seriously by the dialysis team. | 1 | 2 | 3 | 4 | 5 | 6 | 7 | Don’t know | Not applicable |

|  |  | *Always* | |  |  |  | *Never* | | |  |
| --- | --- | --- | --- | --- | --- | --- | --- | --- | --- | --- |
| Q  14 | I worry about who will be available to needle me. | 1 | 2 | 3 | 4 | 5 | 6 | 7 | Don’t know | Not applicable |

|  |  | *Always* | |  |  |  | *Never* | | |  |
| --- | --- | --- | --- | --- | --- | --- | --- | --- | --- | --- |
| Q  15 | I experience problems due to the positioning of the needles once they are inserted. | 1 | 2 | 3 | 4 | 5 | 6 | 7 | Don’t know | Not applicable |

|  |  | *Always* | |  |  |  | *Never* | | |  |
| --- | --- | --- | --- | --- | --- | --- | --- | --- | --- | --- |
| Q 16 | I am nervous before needling. | 1 | 2 | 3 | 4 | 5 | 6 | 7 | Don’t know | Not applicable |

|  |  | *Never* | |  |  |  | *Always* | | |  |
| --- | --- | --- | --- | --- | --- | --- | --- | --- | --- | --- |
| Q  17 | My fistula/graft is assessed before the needles are placed. | 1 | 2 | 3 | 4 | 5 | 6 | 7 | Don’t know | Not applicable |

|  |  | *Always* | |  |  |  | *Never* | | |  |
| --- | --- | --- | --- | --- | --- | --- | --- | --- | --- | --- |
| Q 18 | I have problems communicating with the  dialysis team about my needling. | 1 | 2 | 3 | 4 | 5 | 6 | 7 | Don’t know | Not applicable |

|  |  | *Never* | |  |  |  | *Always* | | |  |
| --- | --- | --- | --- | --- | --- | --- | --- | --- | --- | --- |
| Q  19 | The dialysis team put me at ease during needling. | 1 | 2 | 3 | 4 | 5 | 6 | 7 | Don’t know | Not applicable |

|  |  | *Strongly agree* | |  |  |  | *Strongly*  *disagree* | | |  |
| --- | --- | --- | --- | --- | --- | --- | --- | --- | --- | --- |
| Q 20 | There are things about my fistula/graft that make it difficult to needle. | 1 | 2 | 3 | 4 | 5 | 6 | 7 | Don’t know | Not applicable |

|  |  | *Strongly disagree* | |  |  |  | *Strongly*  *agree* | | |  |
| --- | --- | --- | --- | --- | --- | --- | --- | --- | --- | --- |
| Q  21 | I feel able to tell dialysis team if something doesn't feel right. | 1 | 2 | 3 | 4 | 5 | 6 | 7 | Don’t know | Not applicable |

|  |  | *Strongly agree* | |  |  |  | *Strongly*  *disagree* | | |  |
| --- | --- | --- | --- | --- | --- | --- | --- | --- | --- | --- |
| Q  22 | My needling experience varies greatly from session to session. | 1 | 2 | 3 | 4 | 5 | 6 | 7 | Don’t know | Not applicable |

|  |  | *Always* | |  |  |  | *Never* | | |  |
| --- | --- | --- | --- | --- | --- | --- | --- | --- | --- | --- |
| Q 23 | I experience problems when the needles are removed. | 1 | 2 | 3 | 4 | 5 | 6 | 7 | Don’t know | Not applicable |

|  |  | *Strongly agree* | |  |  |  | *Strongly*  *disagree* | | |  |
| --- | --- | --- | --- | --- | --- | --- | --- | --- | --- | --- |
| Q 24 | Previous bad experiences of needling still affect how I feel about my needling. | 1 | 2 | 3 | 4 | 5 | 6 | 7 | Don’t know | Not applicable |

|  |  | *Never* | |  |  |  | *Always* | | |  |
| --- | --- | --- | --- | --- | --- | --- | --- | --- | --- | --- |
| Q  25 | My buttonhole scabs are removed with as little pain as possible. | 1 | 2 | 3 | 4 | 5 | 6 | 7 | Don’t know | Not applicable |

|  |  | *Strongly disagree* | |  |  |  | *Strongly*  *agree* | | |  |
| --- | --- | --- | --- | --- | --- | --- | --- | --- | --- | --- |
| Q 26 | I get the support I need when new buttonhole sites are formed. | 1 | 2 | 3 | 4 | 5 | 6 | 7 | Don’t know | Not applicable |

|  |  | *Strongly disagree* | |  |  |  | *Strongly*  *agree* | | |  |
| --- | --- | --- | --- | --- | --- | --- | --- | --- | --- | --- |
| Q  27 | The pain relief that I use works well. | 1 | 2 | 3 | 4 | 5 | 6 | 7 | Don’t know | Not applicable |

|  |  | *Worst it*  *can be* | |  |  |  | *Best it*  *can be* | | |  |
| --- | --- | --- | --- | --- | --- | --- | --- | --- | --- | --- |
| Q  28 | How would you rate your overall needling experience? | 1 | 2 | 3 | 4 | 5 | 6 | 7 | Don’t know | Not applicable |

| **If there is any other aspect of your needling experience that you would like to comment on that has not already been covered, please write below.**  *Please use this space for general comments. If this questionnaire has raised any concerns or questions, please contact your kidney team directly.* |
| --- |

*Thank you for completing this questionnaire!*

There are three service-related items that do not form part of the NPREM but that address aspects of care important to needling experience. These items should be collected alongside the NPREM. We recommend that they are collected with any additional personal or clinical information.

**Service-related questions**

S1. I am concerned about the staffing levels at my unit.

*Response scale – Strongly Agree – Strong Disagree/Don’t Know/Not Applicable*

S2. I am aware that pain relief for needling is available to me (for example numbing cream, spray or injection).

*Response scale: Yes/No/ Don’t Know/Not Applicable*

S3. I have a needling care plan.

*Response scale: Yes/No/ Don’t Know/Not Applicable*

## SM14 NPREM Scoring Guidance

Scoring Guide for the NPREM

(Needling Patient Reported Experience Measure)

The Needling PREM consists of 28 items (questions); 27 of these are within five themes of care with one item (Q28) relating to overall needling experience. Participants’ responses are collected on a 1-7 scale or “not applicable” and “don’t know”.

A response of 7 reflects positive patient experience for all questions, except for one question (Q1), where 1 reflects positive patient experience.

Total NPREM scores and NPREM theme scores can be estimated for respondents who have provided an answer (including “not applicable” and “don’t know”) for at least 22 questions (~80%).

Note:

We recommend that a copy of the data is used when scoring the NPREM.

**Initial steps:**

1. Count the number of items (Q1-Q27) containing *any* response (including 1-7, “don’t know” and “not applicable”). If ≥22, proceed to step two**. If <22, you are unable to calculate a score.**
2. **Record the frequency and proportion of “don’t know” and “not applicable”** for each question. Although they are not included in the NPREM score, they relay important information or gaps in knowledge and should be considered and reported. Ensure that this is done prior to calculating the total NPREM or theme scores.
3. **Remove “don’t know” and “not applicable” responses** by replacing with missing values (usually something like “.” or “.a”, depending on the software you use).
4. **Reverse score Q1** ‘Overall, how painful is needling?’ so that less painful needling scores more highly (i.e., replace numbers as follows 1⬄7, 2⬄6, 3⬄5).

**Total NPREM score for each participant**

The total NPREM score is calculated across 27 items, which excludes the overall question (Q28).

Follow these steps to calculate the total NPREM score for each participant:

1. Count the number of items with responses that used the 1-7 scale (N), excluding the overall question (Q28).
2. Add up the total score across all 27 questions (T) excluding the overall question (Q28).
3. Calculate the total NPREM score by dividing the total score (T) by the number of questions responded to (N), i.e., using the formula:

$$NPREM =\frac{T}{N}$$

4. The participant’s overall experience question (Q28) should be reported alongside their total NPREM score.

Example

A participant has responded to the NPREM as follows:

| **Q1** | **Q2** | **Q3** | **Q4** | **Q5** | **Q6** | **Q7** | **Q8** | **Q9** | **Q10** |
| --- | --- | --- | --- | --- | --- | --- | --- | --- | --- |
| 5 | 6 | 6 | 7 | 5 | 4 | D/K | 3 | 7 | 6 |
| **Q11** | **Q12** | **Q13** | **Q14** | **Q15** | **Q16** | **Q17** | **Q18** | **Q19** | **Q20** |
| 6 |  | 7 | 4 | 1 | D/K | 7 | 6 |  |  |
| **Q21** | **Q22** | **Q23** | **Q24** | **Q25** | **Q26** | **Q27** | **Q28 (Overall)** |  |  |
| 6 | 7 | 5 | 5 | N/A | 5 | 7 | 5 |  |  |

*N/A=not applicable*

*D/K=don’t know*

Following the guide above:

1. Reverse score Q1: replace it with the value 3.
2. Total number of items (excluding overall question, Q28) containing a response = 24
3. Items with “don’t know” or “not applicable” responses = 3, these are removed from the data (Q7, Q16, Q25)
4. Number of items (excluding overall question, Q28) with a 1-7 response: N = 21
5. Total score across questions: T = 3 + 6 + 6 + 7 + 5 + 4 + 3 + 7 + 6 + 6 + 7 + 4 + 1 + 7 + 6 + 6 + 7 + 5 + 5 + 5 + 7 = 113
6. NPREM score = $\frac{T}{N}=\frac{113}{21}=5.38$

**Theme scores for each participant**

There are five themes within the NPREM for which a theme (sub-scale) score can be calculated using a similar method as described above. The number of items varies across themes, so please refer to Table 1 (below) to see the required minimum number of responses (1-7, “don’t know”, “not applicable”).

Note:

A new copy of the dataset should be used when calculating theme scores if doing so after the total NPREM score has been calculated, removing any participants where a total NPREM score could not be estimated (those who provided <22 responses) and remembering to reverse score Q1 ‘Overall, how painful is needling?’ (see initial steps 1 and 4).

**Theme scores for each participant**

Each NPREM theme score can be estimated as follows:

1. Count number of items within the theme containing any response (using 1-7, “don’t know”, “not applicable”). Refer to Table 1; if the number of items completed is greater than or equal to the number of items required (M), proceed to step two. If not, you are unable to calculate a score for that theme.
2. Remove “don’t know” and “not applicable” responses by replacing with missing values (usually something like “.” or “.a”, depending on the software you use).
3. Count the number of items within the theme with responses using the 1-7 scale (N).
4. Add up the total score across all questions within the theme (T).
5. Calculate the NPREM theme score by dividing the total score (T) by the number of questions responded to (N), i.e., using the formula:

$$NPREM =\frac{T}{N}$$

*Table 1: Minimum number of item responses required for each theme:*

| **Theme** | **Questions** | **# Items included** | **# Items required to estimate score (M)** |
| --- | --- | --- | --- |
| Communicating with the Team | Q8, Q11, Q13, Q18, Q21 | 5 | 4 |
| My Fistula/Graft and Needling | Q5, Q12, Q20 | 3 | 2 |
| Steps in Needling | Q2, Q15, Q17, Q23, Q25, Q26, Q27 | 7 | 5 |
| Working Together | Q3, Q6, Q7, Q10, Q14, Q19 | 6 | 4 |
| My Personal Experience | Q1, Q4, Q9, Q16, Q22, Q24 | 6 | 4 |
| **NPREM Scale Score** | **Q1-Q27** | **27** | **22** |

Example

Using the same example data as previously (replicated below), scores have been calculated for each NPREM theme.

| **Q1** | **Q2** | **Q3** | **Q4** | **Q5** | **Q6** | **Q7** | **Q8** | **Q9** | **Q10** |
| --- | --- | --- | --- | --- | --- | --- | --- | --- | --- |
| 5 | 6 | 6 | 7 | 5 | 4 | D/K | 3 | 7 | 6 |
| **Q11** | **Q12** | **Q13** | **Q14** | **Q15** | **Q16** | **Q17** | **Q18** | **Q19** | **Q20** |
| 6 |  | 7 | 4 | 1 | D/K | 7 | 6 |  |  |
| **Q21** | **Q22** | **Q23** | **Q24** | **Q25** | **Q26** | **Q27** | **Q28 (Overall)** |  |  |
| 6 | 7 | 5 | 5 | N/A | 5 | 7 | 5 |  |  |

*Communicating with the Team (Q8, Q11, Q13, Q18, Q21):*

1. Total number of items containing a response = 5. This is greater than M=4 (table 1), so a score can be calculated.
2. Items with “don’t know” or “not applicable” responses = 0.
3. Number of items with a 1-7 response: N = 5
4. Total score across questions: T = 3 + 6 + 7 + 6 + 6 = 28
5. Communicating with the team score = $\frac{T}{N}=\frac{28}{5}=5.60$

*My Fistula/Graft and Needling (Q5, Q12, Q20):*

1. Total number of items containing a response = 1. This is fewer than the minimum number of items required (M=2), so the theme score cannot be calculated.

*Steps in Needling (Q2, Q15, Q17, Q23, Q25, Q26, Q27):*

1. Total number of items containing a response = 7
2. Items with “don’t know” or “not applicable” responses = 1 (Q25), these are removed from the data.
3. Number of items with a 1-7 response: N = 6
4. Total score across questions: T = 6 + 1 + 7 + 5 + 5 + 7 = 31
5. Steps in needling score = $\frac{T}{N}=\frac{31}{6}=5.17$

*Working Together (Q3, Q6, Q7, Q10, Q14, Q19):*

1. Total number of items containing a response = 5
2. Items with “don’t know” or “not applicable” responses = 1 (Q7), these are removed from the data.
3. Number of items with a 1-7 response: N = 4
4. Total score across questions: T = 6 + 4 + 6 + 4 = 20
5. Working together score = $\frac{T}{N}=\frac{20}{4}=5.00$

*My Personal Experience (Q1, Q4, Q9, Q16, Q22, Q24):*

1. Total number of items containing a response = 6
2. Items with “don’t know” or “not applicable” responses = 1 (Q16), these are removed from the data.
3. Number of items with a 1-7 response: N = 5
4. Total score across questions: T = 3 + 7 + 7 + 7 + 5 = 29 (remembering to reverse score Q1)
5. My personal experience score = $\frac{T}{N}=\frac{29}{5}=5.80$

*Overall Experience (Q28):*

The participant’s overall needling experience score is the value of Q28 = 5.00.

Therefore, the participant in our example has the following scores:

Communicating with the Team: 5.60

My Fistula/Graft: Missing

Steps in Needling: 5.17

Working Together: 5.00

My Personal Experience: 5.80

Overall Needling Experience: 5.00

Total NPREM Score: 5.38

Once scores have been calculated for each participant, average theme and total NPREM scores can be calculated across your population or within specific groups (e.g., by age), as well as other summary statistics as required.

*©The NPREM can be freely used with reference to Moore, C. et al. 2024. Development and validation of a measure to assess patient experience of needling of arteriovenous fistulas or grafts for haemodialysis access: The NPREM. Under-review. Clinical Kidney Journal.* Any modification will compromise validity and must be clearly stated in reports.

*Appendix Table: NPREM Items and Response Labels*

|  | ***Items by theme*** | ***Response labels*** |
| --- | --- | --- |
|  | *Communicating with the Team* |  |
| Q8 | I am involved as much as I want to be in decisions about my needling. | Not at all - Completely |
| Q11 | Problems during needling are managed well. | Never - Always |
| Q13 | My opinions about needling are taken seriously by the dialysis team. | Strongly disagree - Strongly agree |
| Q18 | I have problems communicating with the dialysis team about my needling. | Always - Never |
| Q21 | I feel able to tell dialysis team if something doesn't feel right. | Strongly disagree - Strongly agree |
|  | *My Fistula/Graft and Needling* |  |
| Q5 | I worry about how long my fistula/graft will keep working. | All the time - Not at all |
| Q12 | I have concerns that current needling practices are harmful to my fistula/graft | Strongly agree - Strongly disagree |
| Q20 | There are things about my fistula/graft that make it difficult to needle. | Strongly agree - Strongly disagree |
|  | *Steps in Needling* |  |
| Q17 | My fistula/graft is assessed before the needles are placed. | Never - Always |
| Q2 | I experience problems when the needles are inserted. | Always - Never |
| Q15 | I experience problems due to the positioning of the needles once they are inserted. | Always - Never |
| Q23 | I experience problems when the needles are removed. | Always - Never |
| Q27 | The pain relief that I use works well. | Strongly disagree - Strongly agree |
| Q25 | My buttonhole scabs are removed with as little pain as possible. | Never - Always |
| Q26 | I get the support I need when new buttonhole sites are formed. | Strongly disagree - Strongly agree |
|  | *Working Together* |  |
| Q3 | I trust the dialysis team when it comes to my needling. | Not at all - Completely |
| Q6 | My needling is rushed. | Always – Never |
| Q7 | I feel that the dialysis team needling me show empathy. | Never – Always |
| Q10 | My needling is done in a way that makes me feel safe. | Never – Always |
| Q14 | I worry about who will be available to needle me. | Always – Never |
| Q19 | The dialysis team put me at ease during needling. | Never – Always |
|  | *My Personal Experience* |  |
| Q1* | Overall, how painful is needling? | Not at all painful - Extremely painful |
| Q4 | My needling experience has improved over time. | Strongly disagree - Strongly agree |
| Q9 | My frame of mind affects my needling experience. | Always – Never |
| Q16 | I am nervous before needling. | Always – Never |
| Q22 | My needling experience varies greatly from session to session. | Strongly agree - Strongly disagree |
| Q24 | Previous bad experiences of needling still affect how I feel about my needling. | Strongly agree - Strongly disagree |
|  | *Overall Needling Experience* |  |
| Q28 | How would you rate your overall needling experience? | Worst it can be - Best it can be |

*Note: Items are numbered by their recommended order, however items Q2-27 may be presented in any order. Responses use a 1-7 Likert scale with labels at endpoints and with ‘Don’t Know’ and ‘Not Applicable’ options.*

**Q1 reversed scored*

**Additional items to include:**

*Free text comments box:*

If there is any other aspect of your needling experience that you would like to comment on that has not already been covered, please write below.

*Please use this space for general comments. If this questionnaire has raised any concerns or questions, please contact your kidney team directly.*

Service-related items not forming part of NPREM but recommended to be collected alongside:

S1. I am concerned about the staffing levels at my unit.

*Response scale – Strongly Agree – Strong Disagree/Don’t Know/Not Applicable*

S2. I am aware that pain relief for needling is available to me (for example numbing cream, spray or injection).

*Response scale: Yes/No/ Don’t Know/Not Applicable*

S3. I have a needling care plan.

*Response scale: Yes/No/ Don’t Know/Not Applicable*
